# Proteostasis failure exacerbates neuronal circuit dysfunction and sleep impairments in Alzheimer’s disease

**DOI:** 10.1186/s13024-023-00617-4

**Published:** 2023-04-21

**Authors:** Christopher Daniel Morrone, Radha Raghuraman, S. Abid Hussaini, Wai Haung Yu

**Affiliations:** 1 Brain Health Imaging Centre, Centre for Addiction and Mental Health, 250 College St., Toronto, ON M5T 1R8 Canada; 2 Taub Institute, Columbia University Irving Medical Center, 630W 168th Street, New York, NY 10032 USA; 3 Department of Pathology and Cell Biology, Columbia University Irving Medical Center, 630W 168th Street, New York, NY 10032 USA; 4 Geriatric Mental Health Research Services, Centre for Addiction and Mental Health, 250 College St., Toronto, ON M5T 1R8 Canada; 5 Department of Pharmacology and Toxicology, University of Toronto, Medical Sciences Building, 1 King’s College Circle, Toronto, ON M5S 1A8 Canada

**Keywords:** Alzheimer’s disease, Sleep, Proteostasis, Autophagy, Unfolded protein response, Tau, β-amyloid

## Abstract

Failed proteostasis is a well-documented feature of Alzheimer’s disease, particularly, reduced protein degradation and clearance. However, the contribution of failed proteostasis to neuronal circuit dysfunction is an emerging concept in neurodegenerative research and will prove critical in understanding cognitive decline. Our objective is to convey Alzheimer’s disease progression with the growing evidence for a bidirectional relationship of sleep disruption and proteostasis failure. Proteostasis dysfunction and tauopathy in Alzheimer’s disease disrupts neurons that regulate the sleep–wake cycle, which presents behavior as impaired slow wave and rapid eye movement sleep patterns. Subsequent sleep loss further impairs protein clearance. Sleep loss is a defined feature seen early in many neurodegenerative disorders and contributes to memory impairments in Alzheimer’s disease. Canonical pathological hallmarks, β-amyloid, and tau, directly disrupt sleep, and neurodegeneration of locus coeruleus, hippocampal and hypothalamic neurons from tau proteinopathy causes disruption of the neuronal circuitry of sleep. Acting in a positive-feedback-loop, sleep loss and circadian rhythm disruption then increase spread of β-amyloid and tau, through impairments of proteasome, autophagy, unfolded protein response and glymphatic clearance. This phenomenon extends beyond β-amyloid and tau, with interactions of sleep impairment with the homeostasis of TDP-43, α-synuclein, FUS, and huntingtin proteins, implicating sleep loss as an important consideration in an array of neurodegenerative diseases and in cases of mixed neuropathology. Critically, the dynamics of this interaction in the neurodegenerative environment are not fully elucidated and are deserving of further discussion and research. Finally, we propose sleep-enhancing therapeutics as potential interventions for promoting healthy proteostasis, including β-amyloid and tau clearance, mechanistically linking these processes. With further clinical and preclinical research, we propose this dynamic interaction as a diagnostic and therapeutic framework, informing precise single- and combinatorial-treatments for Alzheimer’s disease and other brain disorders.

**Graphical Abstract:**

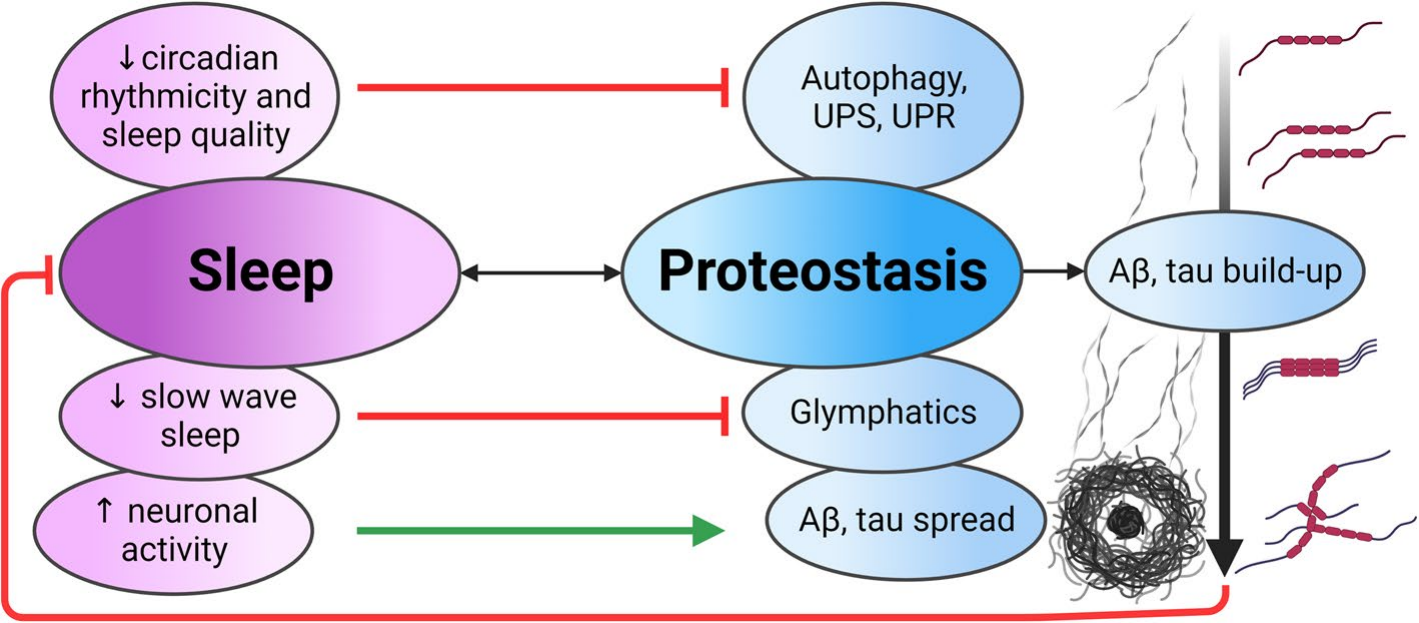

## Background

### Objectives

The objective of this review is to raise attention to emerging evidence of a bidirectional relationship between sleep loss and protein homeostasis (proteostasis) disruption in Alzheimer’s disease (AD), highlighting how this positive-feedback-loop exacerbates neurodegeneration, and AD progression. We explore how sleep impacts the protein life cycle, and vice versa, focusing on post-translational proteostasis: response to misfolded protein, its degradation and clearance from the brain. Clearance of proteinopathy, or lack thereof, is a high priority to help dissect both etiology, diagnosis and treatment of AD and other neurodegenerative diseases (NDDs). We review current pharmacological and lifestyle interventions for sleep, their relation to proteostasis, biomarkers and potential development of preventative, combinatorial and personalized treatment paradigms. Sleep and proteostasis disruptions are key interactive mechanisms underlying rampant proteinopathy in prodromal and symptomatic AD and, therefore, the sleep-proteostasis interplay presents a unique opportunity for disease modification.

### Rationale for the bidirectional sleep-proteostasis relationship

NDDs of aging share a common mechanism of proteinopathy, in that toxic, misfolded proteins accumulate, escalating the seeding throughout the brain, and aggregating in extra- and intra-cellular inclusions [1, 2]. Treatment of proteinopathy must overcome three challenges. Firstly, proteinopathy occurs in decades long prodromal phases, with diagnosis occurring primarily at advanced stages. Secondly, because of extensive proteinopathy at advanced stages, neurodegeneration persists despite interventional treatment. Finally, endogenous processes that normally clear proteins in otherwise healthy individuals are overwhelmed, limiting the efficacy and lasting effects of removing aberrant proteins. In this review we focus on these consequences, possible mechanisms of proteostasis that are overwhelmed in NDDs [1], and reasons why proteostasis fails prodromal to AD.

It is paramount to understand the mechanisms that potentiate β-amyloid (Aβ) and tau spread in AD, especially early events of disease progression. Neurodegeneration and cognitive decline strongly correlate with regional accumulation of tau [2–4], though both Aβ and tau contribute distinct effects on neuronal electrophysiology [5], disrupting behavioral phenotypes, including cognition and sleep. Current models of neuronal network dysfunction in AD ascertain that neuronal hyperactivity occurs as a result of Aβ pathology, while tau is shown to suppress activity [6, 7]. Hyperactivity was observed in layer 2/3 neurons in plaque bearing APP/PS1 mice when compared with wild-type controls; though age-matched rTg4510 transgenic mice expressing aggregated human tau (P301L) without Aβ pathology exhibit significant reduction of cortical activity levels compared to APP/PS1 mice [5]. Combination of Aβ and tau in in vitro entorhinal cortical (EC) slices and in mice demonstrates a suppression in neuronal activity from soluble tau (not dependent on neurofibrillary tangles), and that this effect dominates over the Aβ-induced hyperactivity [5, 8]. These results are supported by evidence in other preclinical models [6, 7, 9, 10]. Tau pathology brings about spatial memory deficits in old but not young EC-Tau mice, wherein excitatory, but not inhibitory, neurons in the medial EC were shown to be vulnerable to tau pathology [11], in line with tau-mediated neuronal suppression. Interestingly, evidence indicates that the presence of Aβ facilitates the effect of tau on neuronal circuit dysfunction in mice [5], and vice versa for the reliance on tau for Aβ-induced hyperexcitability [12–14]. How these effects present in AD patients across progression may rely on differing regional spread of Aβ and tau [3, 15], and is an emerging area of NDD research [6, 7, 16]. For our purposes, we focus on how Aβ and tau impact the neuronal circuitry of sleep [9, 17, 18], and vice versa.

In AD, loss in the quantity and quality of sleep, particularly slow wave and rapid eye movement sleep (SWS; REM), is associated with pathological development [19–21]. These alterations begin early and pose an increased risk for developing cognitive impairment and AD progression [22–25]. Many aspects of proteostasis exhibit sleep-regulated and rhythmic changes in activity. Protein degradation, and clearance from the brain are impaired in AD [1] and intimately linked to sleep [21, 26–30]. In particular, increased periods of neuronal activity potentiate Aβ and tau spread [30–32], reduced glymphatic clearance occurs with SWS loss [27, 29], and dysregulation of the unfolded protein response (UPR), ubiquitin proteasome system (UPS), and autophagic-lysosomal pathway (ALP) results from sleep and circadian disruption [1, 21, 26, 28, 33].

Failed proteostasis, including accumulations of undigested autophagosomes and lysosomes, and proteinopathies like Aβ or tau, cause neurodegeneration in AD-affected brain regions, including the EC, hippocampus, hypothalamus and locus coeruleus (LC) [1, 3, 15, 34]. This neurodegeneration, in turn, impairs neuronal circuitry that regulates memory and sleep. Furthermore, Aβ and tau influence the sleep state and regulate circadian rhythms, respectively [17, 35]. Overall, the dynamic relationship between sleep and proteostasis means impairment of one mechanism exacerbates the other, and together accelerate AD progression.

In sum, sleep loss and proteostasis failure are interactive in AD, involving poor sleep and disrupted circadian rhythmicity which impair biological processes involved in protein clearance. Aβ and tau then feedback to exert direct and indirect effects, via neurodegeneration of sleep–wake controlling neurons, on the sleep–wake cycle. This cycle repeats throughout AD progression; however, we propose that sleep loss and proteostasis dysfunction in the Alzheimer’s prodromal phase exists as a positive-feedback-loop and is a critical driver of disease progression. Although we focus our discussion herein on Aβ and tau, the growing notion of mixed pathology in NDDs [36] and the ubiquity of proteostasis disruption in these disorders [1] demonstrate that the bidirectional sleep-proteostasis relationship impact other protein aggregates involved in NDD.

AD progression is accelerated via a positive-feedback-loop between proteinopathy and neuronal network dysfunction. Given the empirical evidence in the recent decade for sleep as a potent regulator of proteostasis, we postulate the sleep-proteostasis relationship is critical in the early phase of AD: with Aβ and tau accumulation the neuronal electrophysiological signature of sleep becomes impaired, hence contributing to exacerbated proteinopathy.

### Sleep impairment is a risk factor for Alzheimer’s disease

Risk factors associated with increased sleep disorders and NDDs are critical to identifying vulnerable populations and addressing sleep loss. Sleep is a potentially modifiable risk factor for AD [37]. Genetic risk for AD reduces sleep duration, averaging 1.87 less sleep hours per night [38]. A recent meta-analysis concluded that broad sleep impairments (*i.e.*, poor quality, insomnia, under-/over-sleeping, sleep apnea, excessive daytime sleepiness (EDS)) imparts a 1.55 × relative risk for AD; 1.65 × higher for cognitive impairment and most notably for preclinical AD (3.78x) [22]. For example, under-sleeping (< 6 h) for individuals in their 50 s and 60 s and potentially in a preclinical AD stage, increases dementia risk by 30% [39]. Obstructive sleep apnea (OSA), the most common cause of sleep disturbance in adults, poses a significantly high grouped risk for AD (2.37x), and has synergistic detrimental effects with amyloid, tau and neurodegenerative (A/T/N) biomarkers for AD in which hippocampal degeneration driven by AD proteinopathy may exacerbate breathing problems and nighttime apneas [22, 23, 40]. Furthermore, mild cognitive impairment (MCI) associates with significant alterations in sleep across sleep stages, including awakenings throughout the night and decreased sleep efficiency [24]. Cumulatively, this data suggests that sleep disruptions can accelerate AD-associated neurodegeneration most prominently in the pre-symptomatic disease stage, and vice versa.

Genetics can contribute to increased risk of sleep related NDD and AD. Variants of aquaporin-4 (AQP4) which is related to the glymphatic pathway, have been associated with AD pathology in mouse models [41] and in cognitive performance in Parkinson’s disease (PD) [42]. Further dysregulation of AQP4 may be occurring in neurodegeneration (AD and frontotemporal dementia (FTD)), given a higher presence of AQP4 in the cerebrospinal fluid (CSF) [43]. Similarly, a steeper decline in cognitive function was observed in men carrying the apolipoprotein E4 (ApoE4) allele with sleep apnea than ApoE3 [44]. Better sleep consolidation reduces AD risk associated with ApoE4 genotype in older adults without dementia at baseline (mean age ~ 82) and decreases tauopathy/formation of neurofibrillary tangles. This indicates the importance of assessing sleep in ApoE4 + individuals who are at higher risk for AD, and that introducing interventions through sleep could potentially reduce neurofibrillary tangle burden [45].

It is important to recognize that the majority of studies on sleep in AD, in particular those investigating the role of genetic variations such as ApoE, have been conducted in Caucasians, and less is known about other ethnicities with different genetic risk profiles. Recent work has identified an interaction of ApoE4 genotype with OSA in older black adults (average age ~ 70), associating with AD biomarkers including hippocampal volume. This interaction was not observed in white participants [46]. Furthermore, African Americans with at least one ApoE4 allele are significantly more likely to have a shorter sleep duration than African Americans with an E3 genotype [47]. Despite higher sleep disruption in non-race-stratified ApoE4 + participants > 50 years old, the race-stratified effect was not observed in Caucasians [47]. ApoE genotype may also correlate with risk for OSA in Chinese populations [48]. Although the interactive mechanisms of sleep and proteostasis are likely a global phenomenon, future studies should identify the role race and ethnicity play in exacerbating the contributions of sleep loss and proteostasis failure to AD progression.

Sex differences affect an individual’s risk profile for NDD and should be considered in understanding how sleep loss can impact progression to NDDs. AD is seen more frequently in women [49]. While men are more likely to experience sleep apnea, and other sleep disorders are more frequently observed in adult men vs women [50], apneas increase in post-menopausal women [51]. PD and the related synucleinopathy, Dementia with Lewy bodies (DLB) are more frequent in men, yet the increase rates of REM-based disorders may represent an underappreciated risk factor in women who tend to develop the disease later in life [52].

**Fig. 1:**
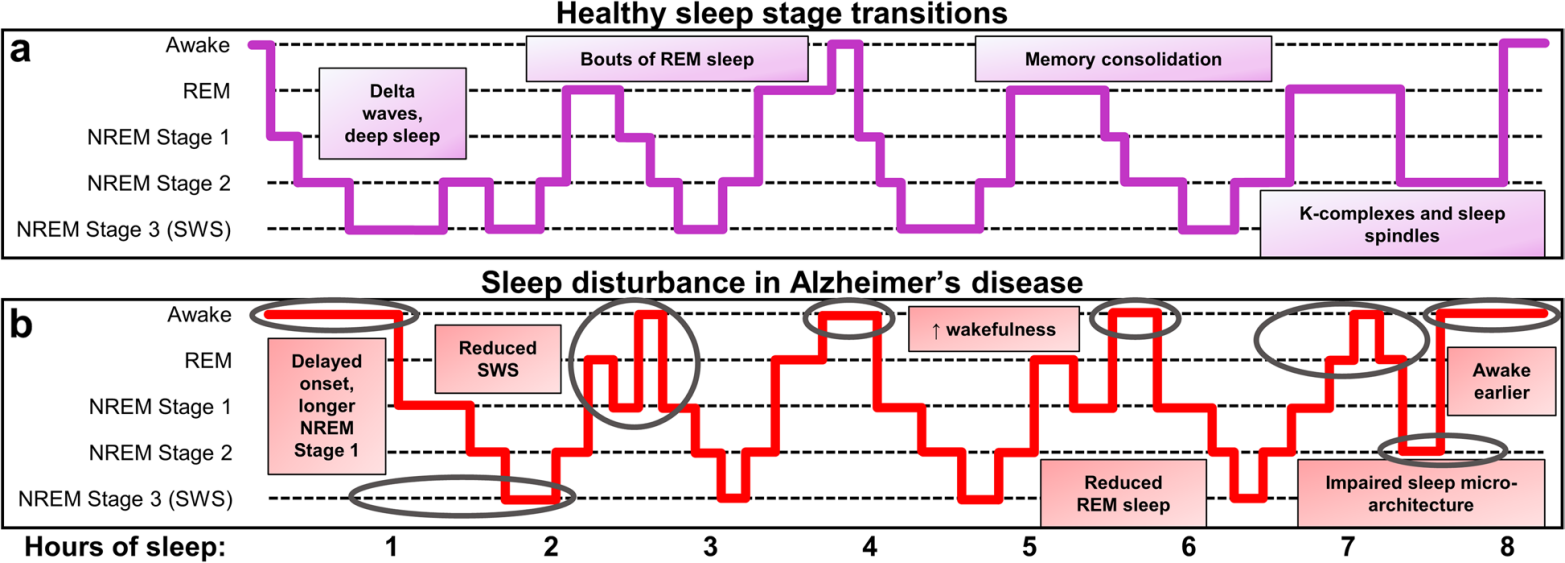
Schematic of sleep disturbances in Alzheimer’s disease. Sleep is subdivided into stages of rapid eye movement (REM) and non-REM (NREM) sleep by signatures of neuronal activity. NREM can be further subdivided into 3 stages; NREM stage 3 is often referred to as slow wave sleep (SWS). **a** In healthy individuals, sleep begins in NREM stage 1, with waning neuronal activity and frequency, which further slows in restorative NREM stage 2 and SWS. SWS dominates early in the sleep cycle with synchronous, low frequency delta waves, whereas transitions to REM sleep occur a few hours after sleep onset, in ~ 90-min cycles. REM sleep electroencephalogram (EEG) is more akin to wakefulness with higher frequency and lower amplitude signals than SWS and dominated by theta waves. Memory consolidation is facilitated by bouts of REM, as well as NREM stage 2, prominent late in the sleep cycle, with characteristic high amplitude K-complexes and high frequency sleep spindles in EEG. In summary, REM and NREM stage 2 and 3 are important in memory consolidation [65–68]; whereas SWS is also critical for toxic protein clearance and to reduce net synaptic strength to dampen aberrant plasticity and preserve a healthy signal:noise ratio of neuronal activity [68, 69]. Individuals who experience sleep disturbances are at a higher risk for Alzheimer’s disease (AD), and, moreover, those with AD exhibit characteristic features of sleep loss. **b** In AD, sleep is disrupted throughout the night, in which there is a delayed onset, longer bouts of non-restorative NREM stage 1 sleep, reduced bouts of SWS, REM and NREM stage 2, as well as increased wakefulness (notable changes compared to healthy sleep are circled). In sum, sleep disturbance poses a significant risk for AD and other neurodegenerative diseases, most prominently through dysregulation of mechanisms that facilitate proteinopathy and cognitive deficits (see Fig. 3). Panels A and B are schematic representations of healthy sleep and common disturbances that occur in AD. Healthy control sleep stages were informed from [68], and the results of the meta-analysis in [22] informed the AD impairments demonstrated in panel B

Exact AD-related sleep disruptions vary across individuals and studies, yet sleep disturbances are common in AD patients. The discussion herein on the relationship of sleep with mechanisms of proteostasis will mainly focus on disturbances of night-time sleep, though the importance of EDS is notable and deserving of attention, including as a factor in circadian arrhythmicity. EDS is common in AD patients, and has been shown to be more severe in patients that have mild DLB, and behavioral variant FTD to a lesser degree [22, 53–55]. This indicates EDS as a common feature among elderly populations and especially in dementia patients. Recent evidence has established a link between Aβ deposition and EDS in healthy adults and elderly individuals without cognitive impairment, making it a potential early predictor of AD [56, 57]. Notably, sleep impairments in aged individuals are intimately linked to impaired cognitive processes [58]. Briefly, AD associates with a multitude of possible night-time sleep disturbances, including longer time to sleep onset, increased time awake and nighttime arousals, less non-REM (NREM) stage 2, SWS, and REM time, and increased NREM stage 1; though many reports indicate the predominance of reduced NREM stage 3/SWS and disrupted slow wave oscillations in AD [59–64]. Figure 1 provides a representative schematic for healthy night-time sleep architecture and staging, its relevance to memory, and comparisons to the impairments that occur in AD.

## Proteinopathy and neurodegeneration accelerate sleep loss

Given the prevalence of sleep disturbances in AD and other NDDs, this section discusses evidence for the effect of Aβ and tau on sleep (though these are expanded upon in subsequent sections in regard to protein clearance, degradation and spread), the relationship of sleep impairment with mixed proteinopathies common in AD patients, and, finally, neurodegeneration of the sleep–wake circuitry in AD.

### Role of Alzheimer’s disease proteinopathy in sleep disruption

#### Direct effects of Aβ on sleep

Both major pathological protein species in AD exert deleterious effects on sleep function [70], and Aβ oligomers interfere with sleep/wake patterns in mice in a dose-dependent manner [71]. Recently, Özcan and colleagues described a direct effect of Aβ on sleep, via injection of oligomers of differential length into zebrafish [17]. Sleep regulation is Aβ oligomer size-dependent, in which short oligomers increase hypothalamic neuronal activity and induce wakefulness via adrenergic and progesteronergic receptor signalling, long oligomers reduce neuronal activity and induce sleep via the prion protein pathway, and very long oligomers have no effect [17]. Conversely, Aβ oligomer injection in mice induced sleep fragmentation (reduced NREM and REM time and increased sleep stage transitions) but this was not observed in prion protein-deficient mouse strains [72], suggesting a complex mechanism for Aβ-sleep regulation. These results may partially explain the broad spectrum of AD-related sleep disturbances, including reduced total sleep time and increased daytime sleep. Furthermore, sleep and circadian rhythm disruptions in AD may trigger this bi-directional control of sleep, in which impaired clearance and metabolism of Aβ causes alterations in diurnal fluctuations [27, 73].

#### Associations of tau with sleep

Multiple recent studies document the relationship of tau with sleep disturbances and electroencephalogram (EEG) abnormalities. In cognitively normal older adults (mean age ~ 73–75), low frequency EEG signal during NREM sleep (1–2 Hz; indicative of delta waves prominent in SWS) exhibit an inverse relationship with AD pathology most prominently with AV-1451 tau positron emission tomography (PET) signal [60, 74], and in AD patients (mild-moderate) sleep–wake disturbances have been shown to correlate with CSF levels of phosphorylated tau [75]. Through an increased neuronal tau release, sleep deprivation in mice and in healthy 30–60-year-old adults compounds this effect with greater brain (in mice) and CSF (> 50% increase in humans) tau [29, 76], in addition to an increase in major AD biomarkers. Higher levels of CSF pT181 and pT217, but not pS202, were observed, with increased levels of non-phosphorylated tau forms at those epitopes [76].

Winer and colleagues assessed associations of tau (^18^F-flortaucipir) and Aβ (^11^C-PIB) PET levels with sleep in older adults (mean age ~ 78), comparing objective (wristwatch actigraphy) and subjective (Pittsburgh sleep quality index: PSQI) measures over 1 week. Objective sleep impairment correlated with greater tau PET in early Braak-related stages, in the EC as well as the medial temporal lobe more broadly, but not with cortical Aβ PET. However, both tau and Aβ are significantly associated with self-reported sleep disturbances, indicating that individuals with more Aβ reported worse sleep quality than they actually had with significant changes in PSQI global and efficiency scores, but not sleep duration. This effect was potentiated by loss of executive function [25]. Whether a subjective underestimation of sleep quality can impact progression to future objective sleep disturbances is an interesting topic for future research; critically, these results indicate the need for EEG, polysomnography (PSG), and actigraphic and accelerometric devices as biomarkers for NDDs (discussed in Sect. "Neurodegenerative disease biomarkers and their relationship to sleep").

Elevated tau has been observed in young individuals with OSA [77], and brain-derived exosomes contain higher levels of total tau, pT181, and Aβ in those with OSA and MCI, compared to OSA alone (age range: 35–65) [78], indicating potentiation of NDD exosome-mediated spread of proteinopathy with sleep disturbances [79–84]. Taken together, these reports indicate an association of tau with sleep that likely contributes to the proteinopathy-sleep bidirectional relationship observed in AD.

Conversely, other studies have demonstrated no changes in total or phosphorylated tau (or Aβ) with 5 days of partial sleep disruption in healthy 20–40-year-olds; total and REM sleep were lost, yet SWS was unimpaired in this paradigm [85], which may have preserved clearance mechanisms such as glymphatics. Overnight interventions in healthy volunteers (aged 35–65), to prevent SWS for one night, also did not show any significant changes in the levels of CSF tau but had elevated Aβ. Poorer actigraphic measures of sleep at home over 6 days associated with higher CSF tau levels in these participants [61]. It is important to note that these two experiments were conducted in young and middle-aged healthy participants, and with increasing age and in NDD, endogenous clearance mechanisms lose efficacy [1], which will contribute to higher protein accumulation after sleep loss.

EEG alpha waves (~ 7–12 Hz) are signatures of rest and quiet wakefulness [74], and have been shown to relate to AD-related tau pathology. In subjective cognitive decline, MCI and AD participants in their 60 s, dampened EEG alpha power and synchronization correlated with increasing levels of CSF total and phosphorylated tau [86], and of CSF phosphorylated tau with a lower peak alpha frequency in the power spectrum of older adults (mean age ~ 70) [87]. When stratified by Aβ (^11^C-PIB) and by tau PET (^18^F-MK-6240) positivity, the peak alpha frequency slowed from ~ 9.5 Hz to ~ 8 Hz in positive groups [87]. In cognitively normal older adults (mean age ~ 75), CSF total and phosphorylated tau, and most notably in those with high p-tau:Aβ ratio, was correlated with an increasing proportion of theta (analyzed in 4–8 Hz) waves, but no changes in delta, alpha or beta frequency bins [88], which may be due to overall EEG slowing. Proteinopathy in the rest-active ‘default mode network’ [89, 90] and tau mediated neuronal suppression [5] may be contributing factors to EEG slowing and impairments in alpha waves. Furthermore, alpha wave effects may stratify by sex: alpha power and total tau negatively correlated in male but not female participants with MCI (mean age ~ 75) [91], and greater resting state alpha EEG activity was reported in female vs. male healthy older adults, as well as in MCI and AD (mean age ~ 69–70) [92]. These results indicate the potential for EEG alpha as a non-invasive AD biomarker, and further research may investigate if impaired alpha wave activity in AD can attenuate the beneficial effects of quiet wakefulness.

The connection of tau with sleep loss and EEG alterations are supported by preclinical models. In tau knockout mice, there is reduced delta power, NREM and total sleep time, and increased state transitions [93]. In P301S (at advanced stages) and rTg4510 tauopathy mice there is reduced delta and theta EEG power associating with sleep alterations [94, 95], and in an FTD-tauopathy mouse model EEG alpha power during the wake-state is decreased [96] indicative of an overall EEG slowing related to tau. Electrophysiological slowing has been shown in rTg4510 cortical neurons, with impairments in NREM sleep UP and DOWN states: prolonging of latency and intervals in UP state and of total DOWN state activity [9]; critically, balance of UP and DOWN states is related to the memory consolidation benefits of sleep [65].

In tauopathy and AD models, sleep deprivation increases tau deposition into paired helical filaments in 3xTg AD mice [97], and tau spread in P301S mice, with hippocampal injection of human tau fibrils, including to LC, involved in arousal in the sleep–wake cycle [29, 98]. Finally, Tg4510 tauopathy model mice exhibit tau inclusions in the suprachiasmatic nucleus (SCN) with arrhythmic expression of PER2 and BMAL1 clock proteins [99]. Tau-deficient *Drosophila* also exhibit circadian rhythm disruption with abnormal activity patterns, impaired neuronal remodeling in pacemaker neurons, and increased circadian clock proteins [35], and *Drosophila* expressing 4R tau have circadian arrhythmicity and disrupted sleep [100]. In sum, tau pathology and loss of function in AD is exacerbated by sleep disruptions, and in turn impairs sleep via circadian arrhythmicity and impairments in sleep-regulating neuronal populations.

### Neurodegenerative disease biomarkers and their relationship to sleep

#### Plasma biomarkers of NDD and of sleep impairment

Plasma biomarkers are a potential method for early detection of sleep-related neurodegeneration, but gold standards for disease diagnosis remain to be established. Headway has been made in AD, where Aβ42, Aβ40, Tau-181, Tau-217 and Tau-231 are all showing promise, along with the inflammatory marker glial fibrillary acidic protein (GFAP) and neurodegenerative marker neurofilament light chain (NfL) [101]. The first three track with sleep disorder changes in CSF levels of Abeta and Tau-181, indicating a direct relationship between sleep disorder and neurodegeneration [102]. Apnea is also associated with increased plasma Aβ42/Aβ40 ratio and phosphorylated-tau [102–104]. Biomarkers of neurodegeneration may represent altered sleep regulation including increased plasma orexin A in plasma in AD [105] and reduced plasma melatonin in Huntington’s disease (HD) [106]. Taken together with the aforementioned increase in AQP4 in CSF [43], this suggests dysfunction of sleep-related pathways in NDDs.

While the majority of biomarkers in relation to sleep dysfunction focus on Alzheimer’s-related proteinopathies, there are other sleep-related biomarkers of disease, including plasma metabolomic [107] and lipidomic [108] profiles. Plasma TNF-α and IL-10 were significantly elevated in REM sleep behavior disorder (RBD) (prodromal to PD) relative to age-matched controls and to decreased IL-6/IL-10 and IL-8/IL-10 levels [109]. TAR DNA-binding protein 43 (TDP-43) and chromosome 9 open reading frame 72 (C9orf72) aggregates are also seen in hypothalamic and SCN neurons that regulate the sleep–wake cycle [110–112]. Development of TDP-43 fluid and PET biomarkers is an active area of research [113–116], and may prove beneficial in amyotrophic lateral sclerosis (ALS)/FTD, limbic-predominant age-related TDP-43 encephalopathy (LATE), and in AD. It is expected that the identification of TDP-43 will take prominence alongside synuclein given that NDDs often have multiple proteinopathies [117–121].

#### AD-associated mixed neuropathology in the interaction of sleep and proteostasis

Table 1 expands our discussion of the role of NDD proteinopathies in sleep disturbances to α-synuclein, TDP-43, fused in sarcoma (FUS) and Huntingtin (Htt). TDP-43, along with tau, is the predominant proteinopathy in FTD and ALS, both of which present with sleep disturbances [122, 123]. Loss of orexinergic neurons and detection of TDP-43 inclusions has been reported in the hypothalamus of ALS patients; though these inclusions likely occur at later stages (III and IV) when there is widespread pathology [111, 112, 124]. Dipeptide repeat inclusions from expansion in C9orf72, a common genetic cause of ALS, have been observed in pinealocytes and SCN vasoactive intestinal polypeptide (VIP) neurons. These neurons regulate circadian rhythms and did not contain phosphorylated TDP-43 inclusions [110], suggesting TDP-43 may not be a significant driver of ALS-associated sleep deficits; whereas C9orf72, ALS-associated motor and breathing impairments could be better indicators of sleep loss in patients [122]. For behavioral variant FTD, there is evidence for a potential relationship of orexin dysregulation and the sleep disturbances in these patients (reviewed in [123]), yet similar to ALS, further work is needed to delineate the roles of TDP-43, tau and FUS pathology, and their homeostasis (see Table 1), on hypothalamic function and sleep.

**Table 1:** Other protein aggregates associated with sleep disturbances

Linkage to proteostasis	Sleep-related disruptions linked to – potential proteostasis impairments	Clinical implications and next steps
**α-synuclein (PD, DLB)**		
α-synuclein (α-syn) present in locus coeruleus and raphe nucleus; PD-related sleep disruptions reviewed in [125] REM sleep behavior disorder (RBD)—physical and often violent dream-enactment, common co-morbidity, and early sign of PD and DLB 43% of RBD patients develop PD, an additional 25% DLB [126], with presence of α-syn aggregates in RBD patients [127]; > 90% of RBD patients exhibit any neurodegenerative disease after 14 years, additionally including MCI and AD [126]		
• ↑ α-syn levels with impairment in UPR, UPS and ALP [1, 125], and spread by neuronal connectivity [128] • Exact linkage of α-syn to glymphatic clearance requires further validation; however, SWS enhancement with ↑ AQP4 channels and ↓ pathology, SWS impairment linked to ↑ α-syn pathology in PD mouse models [129] • AQP4 (+/-) mice with α-syn intrastriatal injections exhibit advanced cortical and striatal α-syn deposition and higher insoluble levels [130]; ligating deep cervical lymph nodes ↓ clearance and ↑ α-syn deposition in A53T mice [131]	**SWS** – reduced AQP4, potentially less α-synuclein clearance by glymphatics **REM** – potentially increased oxidative species with REM loss [125, 132, 133]; potential exacerbation of DLB-related cognitive impairments [68, 69] **Circadian rhythm** – UPR, UPS, ALP impaired, leading to failed degradation of α-synuclein	Synucleinopathy-related RBD is commonly treated with melatonin or Clonazepam (a benzodiazepine) [134]. Other sleep therapies may have success in synucleinopathies, such as orexin antagonists: one clinical trial pilot study for safety and efficacy of Suvorexant for insomnia in PD (ClinicalTrials.gov Identifier: **NCT02729714**, see Table 2) Continued study in role of glymphatic clearance of α-syn, given the relationship between AQP4 polymorphisms and cognition in PD [42] and increased α-syn from sleep disturbances [125] Future preclinical studies may focus on cellular proteostasis of α-syn in relation to sleep disturbances and in sleep-controlling neurons
**TDP-43 (ALS, FTD)**		
TDP-43 inclusions are present in hypothalamus of ALS patients, likely at stage III and IV when TDP-43 is widespread; > 1/3 loss of orexinergic neurons [111, 112, 124] Potential for role of orexin and hypothalamus pathology in behavioral variant FTD [123] ALS patients have disturbed sleep, most commonly from sleep-disordered breathing, including obstructive sleep apnea and hypoventilation. Insomnia, RBD and other sleep impairments can occur as well, reviewed in [122]		
• Small, soluble TDP-43 species are degraded by UPS; insoluble TDP-43 aggregates are broken down by ALP, and can require UPS for full clearance [135]. Impaired glymphatic clearance of MRI tracer in TDP-43 mouse model [136] • ER stress and UPR pathways are involved in ALS, including with the presence of TDP-43, FUS and/or SOD1 [137]	**SWS** – loss in SWS can exacerbate glymphatic deficits seen early in a TDP-43 mouse model [136]; further characterization of the role of glymphatics with TDP-43 and ALS is necessary **Circadian rhythm** – disrupted equilibrium of TDP-43 soluble and insoluble species from UPS and ALP impairment	Further research is needed into the role (if any) that TDP-43 inclusions play in sleep disturbances, potentially through impairment of hypothalamic neurons, or neuromodulatory systems related to sleep (*i.e.*, noradrenaline, serotonin) Cases of FTD-related TDP-43 proteinopathy have rare LC inclusions, whereas FTD-related tau proteinopathy is prominent in LC [138] Hypothalamic impairments in ALS may be more related to metabolic changes, as seen by neuronal loss in the melanocortin pathway in SOD1, TDP-43 and FUS mouse models; upstream serotonin loss observed in the SOD1 mutant mice [139] In sum, evidence indicates hypothalamic metabolic effects are more prominent than hypothalamic sleep deficits from TDP-43, yet there is a potential role of C9orf72 dipeptide repeat inclusions in sleep-controlling neurons exacerbating sleep impairments in ALS (**see **Sect. "Neurodegenerative disease biomarkers and their relationship to sleep"; [110])
**FUS (ALS, FTD)**		
FUS knock-in rat model exhibits increased wakefulness, decreased REM sleep in early dark phase coinciding with upregulation of orexin receptor type 2 [140]		
• FUS regulates transcription of genes involved in autophagy activation and autophagosome formation (shown in vitro), indicating a role in autophagic flux [141] • Impaired with FUS loss-of-function in vitro [141] • Aggregated FUS is mis-localized from nucleus to cytoplasm, and is cleared by ALP and UPS, with evidence suggesting cytoplasmic clearance by the former [142], and nuclear clearance (not exclusively) by the latter pathway [143], reviewed in [144]	**Circadian rhythm and REM** – arrhythmicity in REM sleep and ↑ orexinergic-mediated wakefulness can impact balance of UPS and ALP via impaired clock gene control and sleep loss (see Sect. "Sleep loss dysregulates protein degradation"), leading to ↓ clearance of toxic FUS in nucleus and cytoplasm	Future work can utilize preclinical mouse models to further investigate the regulation of autophagy by FUS. This may elucidate if ALP dysregulation from FUS loss of function in ALS or FTD (when mis-localized to cytoplasm) contributes to proteinopathy, and if this impacts sleep-controlling neurons and has any bearing upon sleep disturbances Further discussion above in TDP-43 section
**Htt (HD)**		
Increased dark-cycle sleep (REM and NREM) but decreased theta and delta power in a HD mouse model starting at the pre-symptomatic stage, indicating likely impaired sleep quality and inability to sustain wakefulness [145]. Circadian arrhythmicity and disrupted clock gene expression in a HD drosophila model [146] HD patients exhibit hypothalamic grey matter reductions and impaired sleep (low efficiency, high arousals, less total sleep time, impaired REM and NREM, etc.) [147, 148]		
• Htt is cleared by autophagy and aggregates impair ALP function. UPS is impaired in HD animal models. Reviewed in [1] • Htt is cleared by glymphatics [149] • ER stress and UPR play a role in HD, mediating Htt toxicity, reviewed in [150]	As described previously for Aβ, α-syn and tau, impaired SWS, REM, and circadian rhythmicity impacts glymphatics, ALP, UPR and UPS, potentially impairing Htt degradation and clearance	Two active, recruiting clinical trials with sleep modifying therapies for HD: 1. Melatonin efficacy for sleep disturbances in HD (ClinicalTrials.gov Identifier: **NCT04421339**) 2. SWS enhancement with an acoustic stimulation sleep headband device in HD, PD and MCI (ClinicalTrials.gov Identifier: **NCT04589182**). Successful promotion of SWS may facilitate Htt clearance via glymphatics [149] Given the impact of ER stress and UPR in HD [150], future preclinical work may investigate potential UPR-sleep interactions in HD models

*α-syn* α-synuclein, *AD* Alzheimer’s disease, ALS Amyotrophic lateral sclerosis, *AQP4* Aquaporin-4, *ALP* Autophagic-lysosomal pathway, *C9orf72* Chromosome 9 open reading frame 72, *DLB* Dementia with Lewy Bodies, *FTD* Frontotemporal dementia, *FUS* Fused in sarcoma, *Htt* Huntingtin, *HD* Huntington’s disease, *MCI* Mild cognitive impairment, *PD* Parkinson’s disease, *REM* Rapid eye movement, *RBD* REM sleep behavior disorder, *SOD1* Superoxide dismutase 1, *SWS* Slow wave sleep, *TDP-43* Transactive response DNA protein-43, *UPS* Ubiquitin proteasome system, *UPR* Unfolded protein response

LATE and DLB are common mixed pathologies in people with AD and present in 1/3 to 1/2 of patients [117–119]. Interestingly, Lewy body pathology but not TDP-43 associates with sleep impairments in AD patients [119]. This is not surprising given the prevalence of RBD in synucleinopathies such as PD and DLB ([126]; Table 1). The presence of these pathologies may confound grouping AD phenotypes as specific Aβ- and/or tau-driven pathologies, but present an exciting avenue for elucidating predictive factors of patient progression or resilience [151], as biomarkers are developed.

#### Sleep biomarkers: EEG, polysomnography, and wearables (actigraphic and accelerometric devices)

Biomarkers of sleep disruption and risk of NDDs like AD and PD remain underappreciated, despite the potential benefit for earlier intervention and reduction of sleep disruption. The most reliable biomarker of sleep disruption is EEG and identification of sleep stage, resting and wake cycles during a sleep cycle. Further daytime sleeping, including episodes of quiet wakefulness may indicate poor sleep quality. Brain regions with highest soluble and deposited Aβ levels, such as ‘default mode network’, exhibit high neuronal activity during quiet wakefulness [89, 90]. Critically, the usage of EEG in NDD and in preclinical research is a promising approach to define predictive biomarkers of sleep and cognitive dysfunction in an array of NDDs, including AD, FTD and PD [152–158].

Newer wearables have advanced sleep detection and may be useful in monitoring RBD, including in individuals prodromal to PD or DLB [159], with wearables providing reasonable measures of I < O index (comparing nocturnal and diurnal motor activity) specificity (89%) and sensitivity (63%), and of wake bouts (sensitivity = 96%), while EEG identified micro-sleep instability better (sensitivity, specificity > 75%) [159]. New wearables have also been compared to PSG/EEG recordings with accuracies of 0.51 to 0.53 in detecting REM sleep, 0.52 in detecting light sleep, and 0.79 to 0.83 in detecting deep sleep [160], indicating that while there is promise, the algorithms on wearables like the Oura ring still require greater accuracy for use as a diagnostic tool. Nevertheless, such instruments hold promise in identification of diseases and health related activity, including PD [161]. The use of wearables shows promise as the algorithm utilizes additional information from the wearer to determine REM and other sleep stages. For example, during REM, autonomic changes include surges in heart rate and blood pressure, irregular breathing and loss of thermoregulation [162], data that can be measured from the device. The development of digital health and wearables are currently in their early stages but show promise for AD, DLB and PD [163, 164], especially as e-biomarkers of sleep quality. The potential is evident, though a possible limitation in such devices is compliance and use by individuals with cognitive impairment.

### Sleep-regulating centers and neurodegeneration

**Fig. 2:**
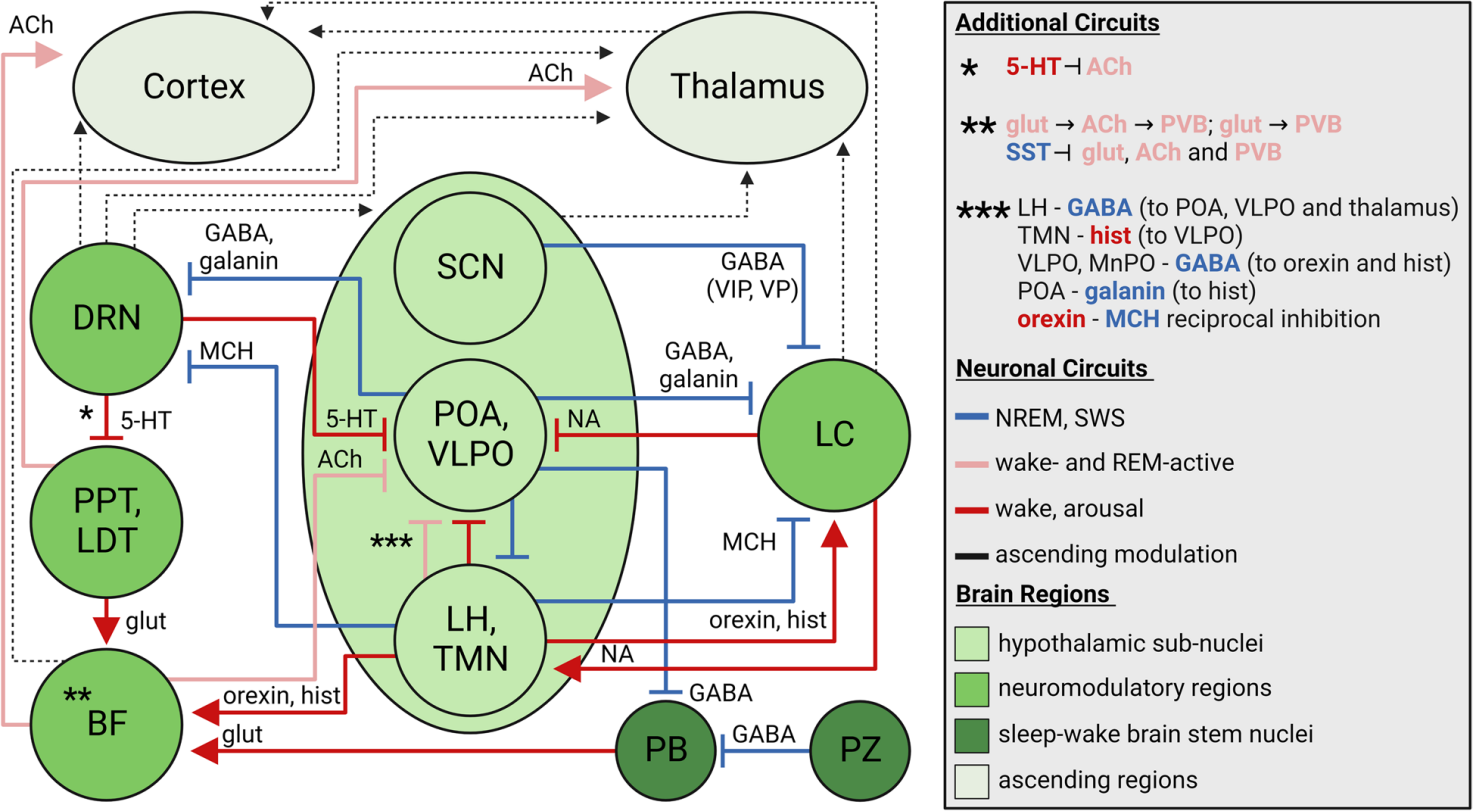
Summary of the sleep–wake circuitry and impact on NREM, REM and wake states. Briefly, neuromodulation from cholinergic (REM-active, wake-active), noradrenergic (wake and arousal) and serotonergic (in general wake-promoting, neuromodulatory sleep-promoting functions) neurons signals to the hypothalamus and ascending pathways to regulate the sleep–wake balance. Hypothalamic orexinergic and histaminergic neurons promote wake, and MCH promotes sleep. GABAergic (VLPO, POA, PZ) and glutamatergic (PB, BF, PPT/LDT) neurons facilitate sleep- and wake-states, respectively; though GABA can be wake-promoting in certain instances. See Sect. "Sleep-regulating centers and neurodegeneration" for further details [165–172]. Regions are not to scale nor laid out anatomically. Arrows indicate activation signal to the efferent region and flat ends indicate inhibitory signal. Synaptic connections are colored by behavioral state: black dashed lines (ascending neuromodulatory activity with broad effects), red (wake and/or arousal), light red (wake- and REM-active), and blue (NREM and/or SWS). Abbreviations: acetylcholine (ACh); basal forebrain (BF); dorsal raphe nucleus (DRN); glutamate (glut); histamine (hist); lateral hypothalamus (LH); median preoptic nucleus (MnPO); melanin-concentrating hormone (MCH); noradrenaline (NA); non-rapid eye movement sleep (NREM); parabrachial nucleus (PB); parafacial zone (PZ); parvalbumin (PVB); pedunculopontine and laterodorsal tegmental nuclei (PPT/LDT); preoptic area (POA); polysomnography (PSG); rapid eye movement (REM); serotonin (5-HT); slow wave sleep (SWS) somatostatin (SST); suprachiasmatic nucleus (SCN); tuberomammillary nucleus (TMN); vasoactive intestinal polypeptide (VIP); vasopressin (VP); ventrolateral preoptic area (VLPO). Created with BioRender.com

In this section we present an overview of the sleep–wake circuitry, focusing on neuromodulatory (noradrenergic, serotonergic, cholinergic), hypothalamic, GABAergic, and glutamatergic sleep-regulation, and relationships to sleep impairments and neurodegeneration in AD, summarized in Fig. 2. Sleep–wake circuitry has been reviewed comprehensively in previous work, including pathways we do not fully capture herein, such as dopaminergic neurons in the ventral tegmental area, other brain stem and midbrain GABAergic and glutamatergic neuronal populations, and the thalamus [165–171]. It is important to note that experiments probing sleep–wake circuitry have been primarily conducted in rodents, with lesions or activation by chemogenetic and optogenetic approaches determining the sleep-state alterations; though the regions and neuronal connections discussed herein are conserved in humans or have human homologues.

#### Locus coeruleus – noradrenergic (wake-active, arousal, REM-inhibiting)

One of the earliest regions affected by hyperphosphorylated tau and neurofibrillary tangles, preceding EC, and hippocampal accumulation, is the LC. LC accumulates tau rapidly between Braak stage 0 and I, with almost all remaining neurons containing tau by Braak stage VI [3, 173–175]. Aβ is seen in later stages in the LC [15], suggesting tau as a selective driver of impairments in the LC. A recent report on the localization and morphology of hyperphosphorylated tau (AT8 +) LC neurons indicates the potential of dendritic spread of tau to LC-connected regions from as early as Braak stage 0, especially from the dorsal LC to neocortex and hippocampus [176]. Through connections with the thalamus, cerebral cortex, basal forebrain (BF), hippocampus and hypothalamus, including inhibition of sleep-promoting GABAergic neurons, LC noradrenergic neurons promote arousal and wakefulness and regulate memory [18, 168, 171, 175, 177], concomitant with the early onset of AD sleep deficits.

Noradrenergic firing is low during NREM and quiescent during REM sleep, which is mediated via GABAergic inhibition from hypothalamic ventrolateral preoptic area (VLPO) and median preoptic nucleus, and hypothalamic galanin- (preoptic area (POA)) and melanin-concentrating hormone (MCH)-neuronal inhibition [168, 170, 171]. Other hypothalamic inputs include orexin, indirect activation from wake-promoting histaminergic neurons, and from the SCN via the dorsomedial hypothalamic nucleus [168, 170]. Noradrenergic outputs include inhibition of VLPO neurons, facilitation of orexinergic-mediated wakefulness, and increased cortical pyramidal neuron excitability [168, 170, 171] (Fig. 2). In sum, LC noradrenergic neurons confer a neuromodulatory tone on sleep-circuitry, promoting arousal and shifting the balance towards the wake state. Noradrenergic vulnerability to tau pathology and autophagic failure [178], as well as its importance in sleep and circadian impairments in AD, is an emerging area of NDD research.

#### Dorsal raphe nucleus – serotonergic (generally wake-promoting and REM-inhibiting, neuromodulatory effects can be sleep-promoting)

Serotonergic signalling has long been known to control sleep–wake circuitry, with broad, neuromodulatory effects throughout a variety of brain regions and neuronal populations (reviewed in [169, 171, 179]; serotonin circuits are of interest in AD (reviewed in [179, 180]. Serotonin neurons are mainly in the dorsal raphe nucleus (DRN) and receive inhibitory afferents which regulate sleep–wake control, including from MCH hypothalamic neurons, and via reciprocal POA(GABA, galanin)-DRN(serotonin) inhibitory activity [168]. Serotonergic neuronal efferents include the thalamus, hypothalamus, cerebral cortex, BF, other brain stem nuclei, and, notably, inhibit REM generating cholinergic neurons in pedunculopontine and laterodorsal tegmental nuclei (PPT/LDT) (Fig. 2). This activity shifts the sleep–wake balance primarily towards wake-state with REM inhibition, though serotonin does exert sleep-promoting effects dependent on the 5-HT receptor subtype [171]. Critically, serotonin activity is reduced through NREM and more so REM sleep [168, 171], similar to what is observed for noradrenaline. In sum, serotonin is a neuromodulator and sleep regulator with intimate linkage to the hypothalamus and cholinergic sleep–wake circuits.

#### Basal forebrain, PPT/LDT – cholinergic (wake- and REM-active) & parabrachial nucleus – glutamatergic (arousal-promoting)

The BF is one of the major hubs of cholinergic neurons which exert broad, neuromodulatory effects. This population of neurons and their efferents are lost in AD forming the core tenet of the cholinergic hypothesis of AD and the therapeutic usage of acetylcholinesterase inhibitors [181]. Critically, BF cholinergic neurons are sleep–wake regulators (as well as regulating other behaviors including memory and attention), with higher activity linked to wakefulness and to REM sleep, and lower activity during NREM [168, 169]. Extensive cholinergic innervation of the cortex indirectly excites pyramidal neurons, with closely linked cortical-BF oscillatory activity especially during wake and REM sleep [169]. This promotes high frequency cortical neuronal activity, and suppresses low frequency, slow delta waves [169]. Notable inputs to cholinergic BF neurons include orexinergic [170], serotoninergic activity with which depolarization or hyperpolarization depends on the 5-HT receptor subtype [171], as well as innervation from glutamatergic neurons in the parabrachial nucleus [166, 168] (Fig. 2). Parabrachial glutamatergic neurons are wake-promoting and provide a major source of arousal from the brain stem [166, 168], and can mediate interoception-related arousal [169]. Other glutamatergic populations have been implicated in the sleep–wake cycle (reviewed in [166, 168, 169]. The parabrachial nucleus-BF-cortex circuit is critical in promoting the wake-state (Fig. 2).

Within the BF, parvalbumin GABAergic and glutamatergic neurons are also wake- and REM-active and interconnected with local cholinergic neurons [168, 182]. Glutamatergic neurons synapse on cholinergic and parvalbumin neurons, and cholinergic connects directly to parvalbumin [168]. Those three neuronal populations are also each inhibited by the neighboring, sleep-promoting somatostatin (SST) GABAergic neurons [168, 182], indicating the complexity of the BF circuitry as a sleep-regulator (Fig. 2).

PPT/LDT cholinergic neurons function similarly to those in the BF, with excitatory efferents on thalamocortical neurons yet notably low cortical innervation [168, 169]. PPT glutamatergic neurons also innervate the BF and have been shown to cause extensive wakefulness upon chemogenetic activation in mice, and more NREM sleep when inhibited [183] (Fig. 2). Furthermore, activation of PPT cholinergic neurons results in reduced EEG slow waves in NREM with an increased light:deep NREM ratio, and activation of GABAergic neurons reduced REM [183].

#### Hypothalamus – orexinergic, histaminergic (arousal, wake-active), and MCH (sleep-promoting, REM-active)

In the hypothalamus, histaminergic (tuberomammillary nucleus), orexinergic and MCH (lateral hypothalamus) neurons are impacted in AD and accumulate tau pathology [18]. These neuronal networks are critical regulators of the sleep–wake cycle balancing arousal (histaminergic), wakefulness (including orexinergic-based reduced REM and SWS), with induction of sleep from REM-active MCH neurons [18, 184, 185], and receive input from neuromodulatory brain stem nuclei (noradrenaline, serotonin and acetylcholine; [168, 169, 171]. Histaminergic and orexinergic wake-promoting neurons are lost in AD patients, yet MCH neurons seem to be preserved and resistant to tau accumulation [18, 186], indicating a misbalance in hypothalamic sleep–wake control, especially considering orexin-MCH reciprocal inhibition [168] (Fig. 2). In AD patients, orexinergic neurons are reduced while CSF orexin is often reported to be increased, suggesting a complex, perhaps compensatory, mechanism for dysregulated sleep–wake signals (loss of nighttime sleep and increased daytime napping) [184, 187]. Notably, orexinergic neurons decrease with age in rodents [188, 189], with hippocampal- and LC-projecting orexinergic innervation depleted in aged rats and macaques, respectively [190–192]. Further implications of sleep- and wake controlling neuronal impairments in AD with regards to influence of tau are reviewed in [18].

Sleep restriction in rodents leads to robust cell loss: LC, hypothalamus, medial prefrontal cortex, and CA1 and dentate gyrus hippocampal layers exhibit significant loss ranging from a ~ 1/4–1/2 the number of neurons [193–196]. Interestingly, LC noradrenergic, and hypothalamic orexinergic neurons decreased with chronic intermittent sleep loss, but not neighboring MCH neurons, and all 3 neuronal populations exhibited reduced density of axonal projections [196]. These effects were sustained after a 4-week recovery period, with the exception of a restoration of MCH projections to baseline levels, indicating a long-lasting, chronic effect that can impact balance of sleep–wake cycles and potentially be further exacerbated in individuals presenting with AD neuropathology [196].

#### Suprachiasmatic nucleus of the hypothalamus (circadian rhythm generator)

The SCN is a structure in the anterior hypothalamus which generates behavioral rhythms via afferents on other hypothalamic nuclei, such as the sub-paraventricular zone and dorsomedial hypothalamic nucleus which then relays to the LC to impact arousal [168, 170] (Fig. 2). The majority of SCN neurons are GABAergic with co-expression of hormones including VIP and vasopressin [168, 169]. The molecular clock and light stimuli modulate the activity of SCN neurons which are more active during day and dampened during nighttime [168]. Critically, SCN neuronal loss [197] and prominent tangle formation with minimal plaque pathology [198], has been documented in AD patients, and the SCN has been linked to circadian disruptions in NDDs including AD, PD and HD [199–201]. Given the vulnerability of SCN VIP neurons to inclusions from C9orf72 expansion [110], ALS and FTD may be considered on this list as well [202].

#### GABAergic circuitry (in general sleep-promoting, NREM-active)

Recent work suggests that beyond the modulatory tone from monoaminergic, cholinergic and orexinergic neurons, the pivotal framework for the sleep–wake cycle arises from fast neurotransmitters such as glutamate and GABA [166, 167]. GABAergic interneurons promote sleep via inhibition throughout the brain [74] but are impaired across AD progression [203]. The VLPO and median preoptic nucleus of POA contain GABAergic neurons that inhibit wake-promoting neurons, including those in the hypothalamus (orexinergic and histaminergic), DRN, LC and parabrachial nucleus. Galaninergic neuronal release from the POA can also inhibit histaminergic and noradrenergic neurons (reviewed in [166, 168]). Input into the VLPO includes inhibition of sleep-promoting neurons from cholinergic, noradrenergic and, less so, serotonergic neurons [168], as well as histaminergic innervation [204] (Fig. 2). Critically, the POA and VLPO in particular, strongly initiate sleep, with lesions of the VLPO in rodents contributing to ~ 40% loss of sleep time [166]. The intermediate hypothalamic nucleus is the likely human homologue of the VLPO, in which AD patients exhibit a loss of galaninergic neurons with the number of neurons significantly associating with the degree of sleep impairment [205]. The parafacial zone of the medulla has also been shown in mice to be a key promotor of SWS and delta wave EEG via GABAergic-mediated inhibition of parabrachial glutamatergic neurons [206]. There are also non-sleep-promoting GABAergic neurons, including the aforementioned BF parvalbumin neurons, as well as lateral hypothalamic GABAergic neurons that can be wake- and REM-active and inhibit sleep-promoting thalamic and POA/VLPO neurons [166, 207–209] (Fig. 2).

Loss of cortical GABAergic tone has been implicated in the impairment of sleep-dominant slow wave oscillatory activity (reduced power without alterations in the oscillatory frequency) in Aβ-driven mouse models, and acute administration of Aβ induces the same electrophysiological impairment [64]. Optogenetic activation of cortical excitatory neurons rescues slow wave oscillations, restores GABA receptor levels and, interestingly, prevents continued accumulation of Aβ plaque [64]. This data demonstrates the dynamic interaction between neuronal circuitry, sleep and neuropathology in AD. During sleep deprivation in mice, hippocampal SST inhibitory interneurons are activated by cholinergic and potentially orexinergic inputs, and thereby suppress local excitatory activity and impair memory consolidation [210]. Nevertheless, inhibitory interneurons demonstrate higher levels of the autophagy activator BAG3, shown to be protective against tau accumulation [211]. It remains to be elucidated how SST autophagy resilience, yet vulnerability to Aβ and tau in AD [211–213], connects to enhanced inhibitory gating which would occur during extended wakefulness [19, 210].

Overall, these sleep-regulating regions and neurons are important to consider in the sleep-proteostasis interaction as their differential vulnerability or resilience to proteinopathy can impact regulation of the sleep–wake balance and may therefore potentiate AD progression and protein accumulation. Future work can examine the connection of these brain regions with bulk protein clearance and these neuronal populations with their susceptibility to failure of cellular proteostasis.

## Sleep impairment accelerates proteinopathy

Sleep is a broad, multicellular phenomena regulated by homeostatic processes that have a cellular/molecular level regulation, wherein a buildup of molecules are proportionate with the duration of time spent awake. An undesirable quantity of such molecules and proteins accumulate in correspondence to an extended duration of wakefulness and when sleep is fragmented [214–218], leading to pathological spread in NDD.

### Sleep loss increases Aβ and tau

#### One night of sleep loss increases Alzheimer’s-related proteinopathy in healthy adults

CSF Aβ and tau are lower in the morning after normal sleep; however, after even one-night of sleep restriction clearance is impaired in middle-aged adults (age range: 40–60) without cognitive impairment, noted by increased morning CSF levels for Aβ42, but not Aβ40 or tau [219]. Conversely, morning total tau in plasma increases by 1.8% from evening levels, and, strikingly, by 17.8% after a night of sleep deprivation in young men (~ 22 years old), with no Aβ plasma changes [220]. Furthermore, Aβ ^18^F-florbetaben PET signal increases after a night of sleep loss in healthy adults (age range: 22–72), especially in the hippocampus [221], indicating relevance to driving AD progression and memory impairments. PET imaging, especially with tau tracers, may prove critical to examining effects of acute sleep loss on proteinopathy. Work from Holth [29] and Lucey [222] and colleagues, demonstrated ~ 30% increased CSF Aβ and > 50% increased CSF tau in healthy adults (30–60 years old) with one-night of sleep deprivation.

After five consecutive nights of partial sleep deprivation in healthy adults (age range: 20–40), a 27% increase in CSF orexin concentrations was observed, without changes in amyloid, astroglial, or neurodegeneration biomarkers. Notably, there was reduced time spent in all sleep stages except for SWS [85], and we speculate that residual SWS when sleep is only partially deprived promotes protein clearance, protecting against increased amyloid which has been observed after total acute deprivation [219, 221]; though the healthy young and middle-aged adults in these studies likely exhibit greater protein clearance efficiency than aged individuals and AD patients.

#### Glymphatic clearance

Brain clearance of a magnetic resonance imaging tracer was reduced immediately following one-night total sleep deprivation in adults (average age ~ 42), and persisted even after sleep was restored [223]. This deprivation likely reduced glymphatic brain clearance which is regulated by neuronal activity, and enhanced during SWS [27, 224, 225]. Glymphatic clearance is one of two mechanisms of interstitial fluid (ISF) clearance from the brain which likely work in consort, the other being the periarterial drainage pathway [226, 227]; though the relationship of glymphatics with sleep is undeniable and therefore is a critical mechanism to consider in the sleep-proteostasis axis and for the clearance of Aβ and tau [27, 29, 225, 228, 229].

**Fig. 3:**
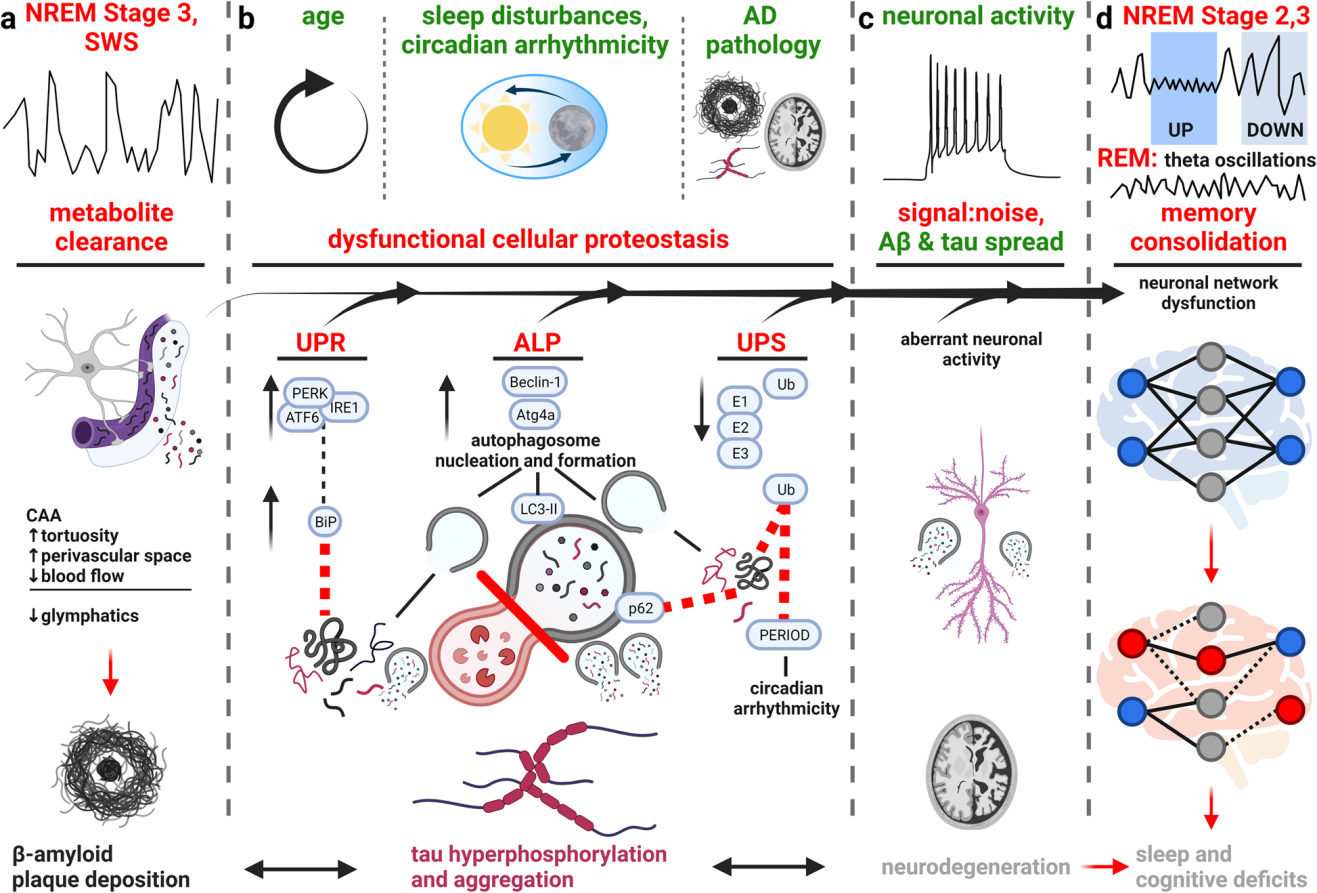
Proteostasis of Aβ and tau is disrupted by Alzheimer’s-related sleep loss, driving proteinopathy, neuronal network dysfunction and cognitive impairment. Sleep is intimately linked to homeostatic processes that control protein accumulation, and when disturbed, can exacerbate and trigger proteinopathy. **a** Shows decrease in SWS and a concomitant decline in the metabolite clearance which is usually highest during SWS. Glymphatics involve brain influx of cerebrospinal fluid (CSF), travelling by bulk flow along periarterial spaces, which crosses the blood–brain barrier (BBB) via an astrocytic AQP4-mediated process, mixes with brain interstitial fluid (ISF), metabolites and solutes, and is cleared along perivenous spaces, driven by vasomotive forces. Bulk CSF/ISF efflux along veins drives metabolite clearance to dural lymphatic systems. Acutely, loss of SWS impairs glymphatic-mediated clearance of β-amyloid (Aβ) and tau, which chronically can feedback in cerebral amyloid angiopathy (CAA), tortuosity, enlarged perivascular spaces, and reduced blood flow, furthering glymphatic disruptions and increasing extracellular protein levels [226, 227]. **b** Sleep disturbance, circadian arrhythmicity, age and AD pathology all impact cellular proteostasis, contributing to an in general overactivation to clear protein; however, in cases of disease, proteostasis is overwhelmed and this activation exacerbates an already damaged system. BiP, and active levels of PERK, IRE1 and ATF6 are increased with sleep loss indicating UPR recruitment, which is insufficient to clear misfolded protein in aged- and diseased-states (indicated by red dashed line). Autophagy activation via Beclin-1 and atg4a, leads to nucleation and upregulated formation of autophagosomes (grey vacuoles), yet with a failure of autophagic flux there is reduced lysosomal (red vacuoles) fusion (indicated by red line). Notably, this can reduce Aβ and tau degradation, impart neurodegeneration through abundant axonal and dendritic autophagosomes, and promote proteinopathy through exosomal release of autophagosomes, as is seen in Alzheimer’s disease (AD) progression. Autophagy is regulated on a circadian cycle, and further impaired when this rhythm is disturbed. UPS failure occurs with disease state contributing to higher levels of intracellular protein that the ALP is unable to compensate for (indicated by red dashed line to p62). Dysregulated UPS-mediated degradation (indicated by red dashed line) of PERIOD proteins (including PER1 and PER2) may further circadian alterations. **c** During periods of prolonged wakefulness, higher frequency neuronal activity without restorative sleep promotes Aβ and tau cell-to-cell spread. Because of elevated synaptic strength, the neuronal signal:noise ratio decreases and synaptic plasticity saturates, leading to non-specific network activity [69]. Without rest, these potentially aberrant neuronal connections, in consort with accumulation of extra- and intracellular uncleared protein, exacerbate neuronal dysfunction, and cognitive processes such as memory can become impaired. **d** Finally, memory consolidation is impaired from loss of REM and NREM stage 2 and 3 sleep, contributing to transient memory loss. Neuronal activity of NREM UP- (*i.e.*, spindles, sharp-wave ripples) and DOWN- (*i.e.*, delta waves, K-complexes) states and REM theta oscillations consolidate memory circuits formed throughout the day [65–68]. Chronically, impairments in proteostasis can progress to rampant accumulation of Aβ and tau in plaques and tangles, respectively, increasing disease spread and neuronal network dysfunction, all of which can further impair sleep and drive cognitive decline. Red text indicates impairments/decreases in AD and sleep disruption, green text indicates increases with AD and sleep disruption. Created with BioRender.com

Glymphatics is a pathway of bulk fluid exchange in which CSF is pumped into the brain from the subarachnoid space, first along the cortical pial arteries. Arterial vasomotive forces move CSF into deeper brain regions in the periarterial Virchow-Robin space, and its transport across the blood–brain barrier (BBB) is mediated by AQP4 channels on the endfeet of astrocytes which encapsulate the brain vasculature. Following CSF-ISF exchange, fluid efflux occurs along the perivenous space to the dural lymphatic system, facilitating the brain clearance of extracellular metabolites and solutes [224]. During SWS, the interstitial space increases by 60% supporting higher rates of glymphatics-mediated Aβ clearance [27], yet during wake or sleep deprivation clearance is reduced with 90–100% higher ISF tau in mice [29] (Fig. 3a). Glymphatic CSF influx significantly correlates with neuronal signals of SWS: low frequency, high-amplitude delta waves and a lower heart rate; whereas a negative correlation was observed with high-frequency beta waves in mice, common in wake state, and no significant relationships with alpha or theta waves, present in wake-state, REM and NREM stage 1 sleep [74, 230].

These results demonstrate the intimate relationship of glymphatic-mediated clearance of Aβ and tau with sleep, and the positive-feedback-loop that can occur as sleep is lost in AD. Mounting vascular impairments occur in AD, including cerebral amyloid angiopathy, vessel tortuosity and rigidity, reduction in cerebral blood flow, and enlargement of periarterial and perivenous spaces; all of which impair glymphatic clearance, and are further impaired by the failure of glymphatic clearance with an abundance of Aβ and tau [226, 227] (Fig. 3a).

Interestingly, diurnal fluctuations in glymphatic clearance show regional differences in the rat brain, with large sleep-associated increases in brain regions involved in circadian rhythm regulation, including the SCN and lateral hypothalamus [231]. Critically, acute sleep deprivation significantly enhances Aβ and tau which can further impair sleep, and lead to persistently impaired protein clearance [219–221, 223].

### Sleep loss dysregulates protein degradation

Mechanisms of protein degradation, in particular autophagic-lysosomal pathway (ALP), ubiquitin proteasomal system (UPS) and unfolded protein response (UPR) are functionally intracellular. However, this does not preclude the effect of these processes on sleep, and vice versa, since impaired proteostasis across a neuronal population or brain region can exert network effects [232]. For example, Fu and colleagues demonstrate cell- and region-specificity of EC excitatory neurons to autophagic deficits in human brain tissue, facilitating their vulnerability to tau [211] and recently, failure of neuronal autolysosome acidification was identified as a precursor to Aβ plaque formation in AD patients and mouse models [233]. These neurons have impaired autophagy, but considering the specificity of this event across a neuronal population, the effect can cause a spread of pathological proteins at the network level, including during sleep disruption. Individualistic and synergistic effects of Aβ and tau on neuronal network dysfunction [5, 6], and cell-to-cell spread of proteinopathy [234] further compound the effect of proteostasis failure throughout the brain. We propose the interactions of sleep and circadian rhythm with autophagy, UPS and UPR are critical mechanisms driving Aβ and tau proteinopathy from early AD stages. Figure 3 brings out the relevance of the interaction of sleep with cellular proteostasis mechanisms in AD (Fig. 3b), and how mounting pathology impacts neuronal circuitry (Fig. 3c) and cognitive decline (Fig. 3d).

#### Autophagy and sleep impairment

One of the earliest features of disrupted autophagic flux in AD is an abundance of autophagosomes, which accumulate in neuronal cytoplasm, axons, and most prominently in dystrophic neurites. There is a failure to clear autophagosomes, preventing degradation of Aβ, tau and other proteins within [235]. As autophagic deficits mount, so does neuronal damage, contributing to AD progression [1, 34, 236]. Furthermore, uncleared autophagosomes and autolysosomes accelerate the cell-to-cell spread of Aβ and tau via exosomal release [79–84]. Interestingly, disrupting sleep recapitulates AD-related autophagic dysfunction [237–239], and knocking out autophagic function is sufficient to recapitulate a neurodegenerative phenotype [240, 241].

Genetic manipulation of autophagic flux in drosophila has demonstrated the bidirectional link between sleep and autophagy. During sleep, autophagosomes drop compared to wake periods. Blocking autophagosome formation increases sleep, whereas blocking autolysosomal degradation decreases sleep [239]. In mice, acute sleep deprivation causes autophagosomes to accumulate in hippocampal neurons, with increased expression of LC3B, Beclin-1 and p62, indicative of recruitment of autophagic processes [1, 237]. Autophagic flux is disrupted in mice with chronic sleep fragmentation, with increased autophagosomes, endosomes, and number and size of intracellular lysosomes [238]. This is accompanied by spatial learning and memory impairments after acute and chronic sleep deprivation [237, 238]. Therefore, autophagic recruitment without lysosomal fusion and protein degradation occurs with mounting sleep impairments, furthering proteinopathy and disease progression (Fig. 3b, ALP).

Interestingly, Xie and colleagues report cortical and hippocampal Aβ intracellular accumulations in chronic sleep fragmented wild-type mice, akin to observations in AD models [29, 238, 242]. The authors attribute this to a failure of normal amyloid precursor protein (APP) processing via the endosome-autophagosome-lysosome (EAL) pathway in which reduced flux through the EAL pathway decreases APP trafficking and clearance, facilitating amyloidogenic processing and Aβ accumulation [238, 243, 244]. These results are exciting as they demonstrate a potential mechanistic linkage between sleep loss and AD proteinopathy.

#### Circadian rhythm of autophagy

Circadian rhythms and the sleep–wake cycle impact gene expression [217] and are linked to AD pathology. AD patients exhibit circadian arrhythmicity, measured in the clinic by activity/rest cycles, exhibiting delayed cycle phases and lower-amplitude peaks (reviewed in [245]). In aged mice with a knock-out of the biological clock gene *Per1*, autophagy is impaired, and Aβ42 and presenilin levels are higher [246, 247]. *Per* genes (including *Per1* and *Per2*) encode the PER1 and PER2 proteins, often referred to collectively as PERIOD protein, which are essential regulators of the timing of circadian oscillations [248–251]. Critically, the circadian clock regulates expression of autophagic genes [26, 252–254], including transcription factors Nr1d1 and C/EBPβ which promote rhythmic expression of autophagy activators, including Beclin-1 and atg4a [26, 252], triggering autophagosome formation. In the mouse hippocampus, autophagosome-related LC3-II, but not cytoplasmic LC3-I, exhibits a circadian rhythmicity, indicative of higher autophagosome formation and autophagic flux in the sleep-associated light phase as opposed to the dark phase [28]. This differs from autophagosome over-accumulation in AD, in which there is a failure of autophagic flux and protein clearance. Notably, the ALP is regulated on a circadian cycle, and arrhythmicity in AD can further the already present autophagic deficits, akin to what occurs with sleep disturbances (Fig. 3b, ALP).

After chronic sleep fragmentation in mice, overall light phase peaks and dark phase lows of hippocampal autophagic flux remain, but the rhythmicity is impaired with abnormal changes within each phase [28]. Acute recovery sleep is insufficient to reverse these changes, and triggers increased Beclin-1 expression during the sleep-associated light phase [28]. Two conclusions can be drawn from this work to frame our understanding of the sleep-proteostasis interaction in AD. Firstly, sleep loss disrupts circadian rhythmic-regulation of autophagic flux, leading to increased Aβ and tau aggregation. Secondly, these deficits persist after recovery, including the continued, aberrant activation of an already overwhelmed autophagic-lysosomal system in AD by Beclin-1, indicating chronic complications for AD progression.

#### Unfolded protein response (UPR) is impaired with age, after sleep loss, and in Alzheimer’s disease

Normally tasked to clear misfolded protein, the UPR is overwhelmed in NDDs, and further exacerbated when sleep is disrupted [1, 33]. Protein kinase RNA-like ER kinase (PERK), inositol-requiring enzyme 1α (IRE1), and activating transcription factor-6 (ATF6α and β) are three crucial proteins that help initiate UPR and from which the binding immunoglobulin protein (BiP) is unbound in response to misfolded proteins. Chronic activation of UPR, especially on the PERK branch, occurs in the brains of AD patients and other tauopathies. AD brains have a heightened expression of UPR activation markers, including phosphorylated PERK, eukaryotic initiation factor 2α (eIF2α), and IRE1, as well as BiP, which correlate with Braak stages [255–258], suggesting that UPR has a bearing on AD pathology at an early stage. There is an increase in UPR in tauopathies; and UPR associates with early hippocampal tau pathology in these disorders [259]. Furthermore, the same neurons and glia that display abnormal tau phosphorylation levels show a corresponding increase in markers of UPR activation as well, corroborating their linkage [259].

UPR chaperones are upregulated during wakefulness, or when sleep is deprived, to help mitigate endoplasmic reticulum (ER) stress by clearing misfolded proteins and reducing protein translation [21, 33]. The ER chaperone BiP binds to misfolded proteins to prevent aggregation and promote re-folding. In the mouse cortex, BiP levels increase progressively as sleep is deprived [216], as a compensation to accumulation of uncleared protein. This phenomenon has been observed in drosophila as well, in which BiP levels rise during sleep loss and fall towards baseline throughout recovery sleep [218]. Increasing normal BiP expression, or of a dominant negative form which decreases BiP function, prolonged or reduced sleep recovery after sleep deprivation, respectively [218]. BiP overexpression slows UPR function [218, 260], which is suggestive of how chronic sleep disruption in AD patients can lead to a state in which the UPR is overwhelmed by abundant misfolded protein (Fig. 3b, UPR). These genetic manipulations had no effect on baseline sleep in the flies [218]. These data are indicative of a parallel UPR activation to mitigate protein accumulation during sleep impairment that is intimately linked to sleep behavior [216, 218].

Interestingly, acute sleep deprivation in young mice promoted protein clearance and reduced translation via UPR, but in aged mice led to pro-apoptotic signalling [261]. Sleep and protein quality control are both impaired with aging [33, 262] resulting in reduced efficiency of refolding aspects in UPR [261, 263–265]. This is evident from the undersupply in chaperone proteins in age related diseases and in aged wild-type rodents [261, 266, 267], corroborating the impact that sleep quality and protein homeostasis have on each other [33]. UPR is activated in orexinergic and noradrenergic wake active neurons with increases in phosphorylated-PERK. This occurs to a greater degree in aged mice correlating with a decline in orexinergic and noradrenergic neuronal activity, with nuclear translocation/activation of CCAAT/enhancer binding protein homologous protein (CHOP) [268]. CHOP is known to signal apoptosis in response to ER stress [269, 270] and to mediate sleep apnea/hypoxia-related cellular stress and injury [271, 272].

The stress response and quality control of the protein homeostatic system becomes dysfunctional with almost all tissues of aged candidates [261, 263, 265], reviewed in [273], demonstrating a complex interaction in AD and other neurodegenerative disorders wherein age, impaired proteostasis and sleep exert individual and synergistic impacts on disease progression. Importantly, UPR chaperone-mediated treatment restores aging related sleep and cognitive impairments in mice [274]. Another UPR therapeutic target for NDDs is eIF2α which attenuates global protein synthesis rates critical for memory and neuronal function, when it is phosphorylated by phosphorylated-PERK; phosphorylated-eIF2α levels are elevated in Alzheimer’s disease and other NDDs [275, 276]. In vitro screening assays of clinically suitable therapies identified that the anti-depressant trazodone hydrochloride, and one other compound (dibenzoylmethane), reversed the effects of phosphorylated-eIF2α on protein synthesis. In vivo treatment of either compound normalized translation and was neuroprotective in prion and tauopathy models [276]. Critically, trazodone can be used to treat insomnia and improve sleep maintenance [277], further indicating the therapeutic potential of the sleep-proteostasis interaction (reviewed in [278]).

Persistent upregulation of UPR is detrimental after extensive proteinopathy and sleep loss, interrupting protein synthesis, promoting neurodegeneration and exacerbating defective sleep-proteostasis positive-feedback-loop [1, 21]. If proteins cannot be appropriately folded in the ER lumen, site of protein synthesis and packaging, proteins are directed to the proteasome to avoid aggregation [279, 280]. Finally, mild ER stress can precondition a neuroprotective mechanism for UPR via recruitment of autophagic processes [1, 281]. However, with prolonged stress like chronic sleep deprivation, UPS, and ALP recruitment to compensate for UPR failure may further overwhelm cellular proteostasis.

#### Ubiquitin proteasome system (UPS) and sleep impairment

The interaction between UPS and sleep disruptions is not as well defined as with autophagy and the UPR. There is evidence that OSA reduces proteasomal activity via intermittent hypoxia [282], which contributes to proteasomal dysfunction after neurodegeneration [1, 283]. With mounting UPS deficits, ubiquitination and degradation of PER1 and PER2 proteins, biological clock proteins and circadian length-regulators, become unbalanced, lengthening circadian rhythms [284] (Fig. 3b, UPS). Tau accumulation is intimately linked with proteasomal dysfunction [285]; although research is needed to elucidate if there is a defined tau-UPS-sleep component contributing to proteinopathy and sleep loss in AD. Importantly, dysfunction in UPS causes compensatory activation of autophagic processes, which are already aberrant in AD [1, 286, 287], leading to chronic deficits in the sleep-proteostasis axis.

## Future directions to restore the sleep-proteostasis axis

### Disease model: Alzheimer’s disease proteinopathy spreads via neuronal activity

The hypothesis that proteinopathy spread in AD occurs as a repercussion of neuronal activity is gaining traction recently [4, 288, 289], with several groups coming to the same conclusions using different experimental modalities. Mechanistically, tau release into the extracellular space is enhanced by neuronal activity, where it can spread and seed pathology via cell-to-cell propagation [32, 290, 291]. This is observed for Aβ as well [292–294]. In humans, higher hippocampal activation positively correlates with Aβ PET levels, and associates longitudinally with declining memory performance [295].

Neuronal activity is most suppressed during SWS. Therefore, sleep loss, increased arousals, and more time awake in AD is contributing to longer periods of high neuronal excitability, and Aβ and tau spread. Disruption of NREM slow wave activity was proportionate with the increase in the levels of Aβ in medial prefrontal cortex of cognitively-healthy older adults [296]. Similarly, an increase in tau correlates with diminished delta power (1–4 Hz) [60]. These studies corroborate the evidence for linkages between sleep, neuronal circuit disruptions, and Aβ and tau (Fig. 3c). The aforementioned observation of increased morning CSF Aβ levels when arousal was induced specifically during SWS in healthy humans [61] was likely driven by increased ISF Aβ during periods of higher neuronal activity (along with extracellular tau release) [61, 290, 291, 293, 294, 297]. ISF Aβ concentration is greater during wakefulness and lesser during sleep [297], with > 20% increased ISF Aβ and lactate levels, a marker of neuronal activity, during the dark- as opposed to the light-period in hippocampi of young Tg2576 mice [294]. Physiological neuronal activity dynamically regulates ISF Aβ levels in vivo indicating region-specific vulnerability given the proclivities of plaque deposition in ‘default mode network’ [294].

Inhibitory neurons dominate in the dampened, deep sleep state. Brainstem neurons balance the reciprocity between NREM and REM (mostly GABAergic, with some evidence for glutamatergic regulators), indicating the importance of SWS and NREM sleep for switching to the REM state (reviewed in [168]). Serotonergic and noradrenergic tone is diminished through NREM and quiescent in REM sleep. The inhibition of serotonin and noradrenaline, including from hypothalamic MCH neurons and POA GABAergic and galanin neurons, are also implicated in the emergence of and maintenance of REM sleep [168, 170, 171]. Critically, higher rates of neuronal activity associate with increased APP processing, which in a neurodegenerative environment with chronic stressors (*i.e.*, oxidative stress) can further shift the balance towards amyloidogenic vs. non-amyloidogenic processing [298, 299]. This is supported by evidence suggesting increased Aβ production during sleep impairment drives higher CSF Aβ levels in healthy adults (age range: 30–60) [222]. Therefore, we can posit that higher rates of Aβ generation with loss of deep and REM sleep is an additional mechanism linking proteinopathy to sleep and neuronal activity, especially in noradrenergic and serotonergic neurons that are usually in a low-activity state during sleep, especially in REM sleep.

In an AD rodent model overexpressing human APP pan-neuronally and tau in the EC, the presence of Aβ accelerates tau accumulation and spread to the hippocampus, and causes EC excitatory neuron hyperactivity, with higher firing rates. Blocking higher rates of neuronal activity subsequently dampened Aβ and tau accumulation and spread [30]. This work suggests a disease model in which Aβ-induced hyperexcitability potentiates tau misfolding and aggregation, leading to tau-induced neurodegeneration and neuronal silencing [5, 30] (Fig. 3c). High rates of neuronal activity, as occurs with sleep impairments and in AD, decreases signal:noise, contributing to non-specific and potentially aberrant synaptic connections and plasticity. This forms part of the synaptic homeostasis hypothesis of sleep, which states that the restful quality of sleep derives from maintaining a balance in synaptic energy usage, stress, metabolic demand, plasticity and activity, which promotes healthy neuronal and cognitive functioning (reviewed in [69]; Fig. 3c).

It was recently demonstrated that neuronal activity inversely correlates with BBB efflux receptors and core circadian clock gene expression in endothelial cells, including PAR bZip and *Bmal1*-dependent signalling [300]. The authors propose a neuronal activity-dependent suppression of BBB efflux in the wake-state, and potentiation in the sleep-state [300], which may contribute to failure of Aβ clearance through these mechanisms in AD, when sleep is impaired and hyperexcitability occurs [5, 19, 301, 302].

Given the significance of neuronal activity in impacting proteinopathy, a recent study demonstrates that APP transgenic mice exhibit reduced neuronal activity in thalamic reticular nucleus (TRN) bringing about increased sleep fragmentation and reduced SWS in comparison to non-transgenic littermates. A selective activation of TRN using excitatory DREADDs rescued the aforementioned deficits and thereby, the amyloid plaque load in hippocampus and cortex [303]. 

Overall, these reports demonstrate that neuronal activity modulates the production, spread, clearance, and interaction of Aβ and tau (Fig. 3c), contributing to the neuronal network dysfunction and cognitive decline that occurs across Alzheimer’s progression. Sleep exhibits distinct neuronal electrophysiological signatures, including timed switching between UP-state thalamic spindles (~ 10–15 Hz) and hippocampal sharp-wave ripples (~ 100–250 Hz) and DOWN-state cortical delta waves (~ 1–4 Hz) and K-complexes (low frequency, high amplitude) during NREM sleep, and hippocampal theta oscillations (~ 4–10 Hz) during REM sleep. The coordination and timing of sleep-related neuronal activity increases the signal:noise ratio, strengthening memory-related synaptic connections (typically newly formed), and conferring memory consolidation ([65–68]; Fig. 3d). In sum, these results suggest modulation of neuronal activity, and in particular sleep-related electrophysiology, as a potent therapeutic strategy for AD to improve cognition and promote proteostasis.

### Probing and treating the sleep-proteostasis axis

**Fig. 4:**
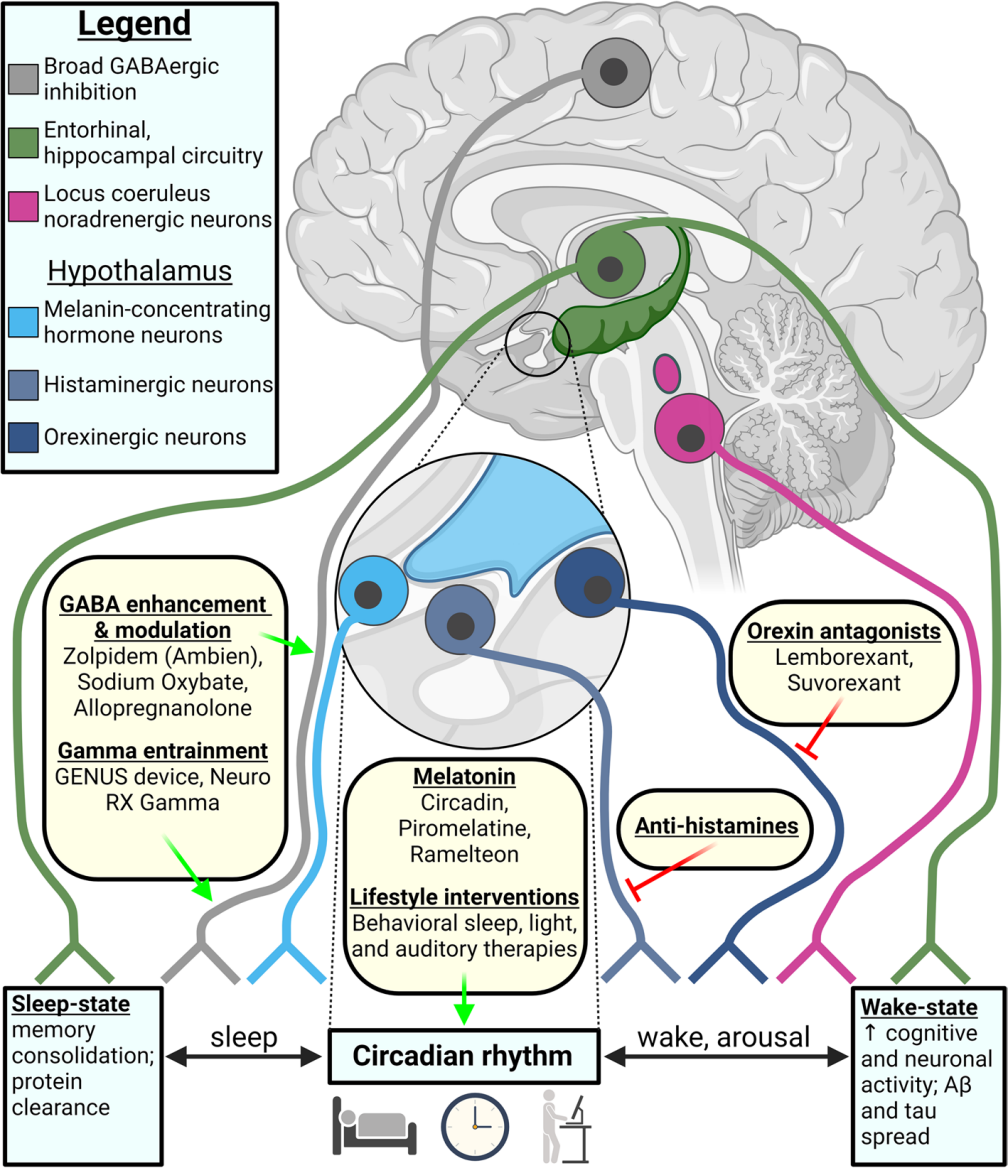
Neuronal control of the sleep–wake cycle and therapeutic targets for sleep restoration in Alzheimer’s disease. The sleep–wake cycle is controlled by neuronal populations vulnerable in Alzheimer’s disease (AD). Circadian rhythmicity is mediated by hypothalamic neuronal activity and melatonin release from the pineal gland, normally maintaining a healthy sleep–wake cycle. Sleep is promoted by activity of melanin-concentrating hormone (MCH)-neurons in the hypothalamus and broad (including cortical, hippocampal, hypothalamic) GABAergic inhibitory signals. In the sleep-state, protein clearance and memory consolidation, mediated by entorhinal-hippocampal circuitry, are enhanced. Conversely, wakefulness and arousal are promoted by activity of histaminergic and orexinergic neurons of the hypothalamus, and noradrenergic neurons in the locus coeruleus. In the wake-state, cognitive and memory processes (mediated by entorhinal-hippocampal circuitry) occur with higher rates of neuronal activity, which potentiates Aβ and tau spread. Therapeutics to enhance sleep in AD present a unique opportunity to simultaneously improve the behavioral phenotype and reduce proteinopathy by improved proteostatic clearance. Enhancement of GABA signalling with pharmacological and non-pharmacological interventions may broadly improve network dysfunction in AD, for memory and sleep circuits. Notably, gamma entrainment is a novel and non-invasive strategy. Sleep promotion and balancing of circadian arrhythmicity can be accomplished via supplementation of the biologically active hormone melatonin, or non-pharmacological lifestyle interventions, including behavioral, light, music, and other auditory therapies. Pharmacological antihistamines and orexin antagonists decrease wake/arousal-signals and promote sleep. Potential exists for targeting of additional neuronal pathways to promote sleep, including noradrenergic signaling which is affected early in AD; the α2 adrenergic receptor agonist dexmedetomidine has been tested (see Table 2), but is more suitable as a sedative than therapeutic. Furthermore, the antidepressant trazodone has potential for improving sleep in AD acting through neuromodulation of serotonergic, adrenergic, histaminergic, and cholinergic pathways, as well as modifying the UPR. Future work is necessary to characterize and discover new sleep- and proteostasis-targeted therapies in AD. See Tables 2 and 3 for sleep-related AD clinical trials and their relevance to proteostasis. Created with BioRender.com

We sought to assess sleep therapies that have reached clinical trial stages for AD. A detailed review and meta-analysis of sleep therapies in dementia has been recently conducted [304]; whereas our purpose was to collate those therapies that demonstrate the potential for repurposing as modifiers of the sleep-proteostasis axis. Therapies were found via a search in clinicaltrials.gov (February 2023): Condition or Disease: Alzheimer’s disease; Other terms: sleep; Study Type (interventional) and was manually restricted: removed non-interventional studies, removed behavioral studies not directly sleep-associated, removed Aβ-targeted trials (*i.e.*, immunotherapies, β-secretase inhibitors, anti-aggregants), already approved for AD, or were not related to sleep modulation. This search yielded 108 intervention trials, 44 using pharmacological or nutritional/dietary supplements (Table 2) and 64 non-pharmacological treatments (Table 3). Their relevance to proteostasis (Tables 2 and 3) and mechanisms (Fig. 4) are documented.

**Table 2:** Pharmacological sleep interventions in clinical trials for Alzheimer’s disease

**Relevance to proteostasis**	**Approval, usage**	**# Trials (# Ongoing)** **Clinicaltrials.gov Identifier /** trial results (if reported)
**Orexin antagonists **(inc. lemborexant, suvorexant, seltorexant)**—Orexinergic signaling **promotes wakefulness; blocking the orexin receptor promotes sleep (see Fig. 4) [18, 184]		
• While not a major contributor, orexinergic impairments occur in AD • Vulnerable to tau pathology and likely potentiates AD-related sleep deficits • Orexin-A neuropeptide regulates (enhances or decreases depending) autophagy via OXR1 signalling [305–307]—potential for synergistic sleep-proteostasis benefits of orexin antagonism in AD • Potential Experimental Use—treatment can be used to assess how tau imparts cell autonomous autophagic deficits, and if rescuing orexin function and/or promoting sleep improves tau clearance	Insomnia (lemborexant, suvorexant), sleep disturbances in AD (suvorexant)	**4 (2)** **NCT02750306** – Phase 3, suvorexant, insomnia in AD; significant improvements in total sleep time vs. placebo; results published: [308] **NCT03001557** – Phase 2, lemborexant, irregular sleep–wake rhythm disorder and mild-to-moderate AD; primary outcomes assessed a wide variety of sleep parameters; overall, no strong benefit on sleep, some significant reduction of restlessness in treated groups vs. placebo No results—**NCT04629547**^b^ No results—**NCT05307692**^b^
**Melatonin **(inc. circadin, piromelatine, ramelteon)**—**Hormone balances circadian rhythmicity [309–311] (see Fig. 4)		
• Hormone released from the pineal gland • Mediates regulation of circadian rhythms and promotes sleep • Potential Experimental Use—used to probe interaction between sleep/biological clock disruptions with autophagic deficits (see Fig. 4) • Melatonin in AD may simultaneously improve sleep and proteostasis	Oral/dietary supplement for sleep	**5 (1)** **NCT00940589** – Phase 2, circadin (prolonged-release melatonin), mild-to-moderate AD; cognition (↑MMSE, ↔ ADAS-Cog) improved sleep (↑PSQI), and behavior (↑IADL) [312] **NCT00000171** – Phase 3, melatonin, AD; primary sleep measures not statistically significant (nocturnal sleep time), trend for nocturnal sleep improvements [313] **NCT02615002** – Phase 2, piromelatine (multimodal melatonin receptor and serotonin receptor agonist), AD; GWAS identified a 6 SNP cluster in chromosome 2q12 which predicted efficacy of treatment: non-carriers improved on ADAS-Cog14 and PSQI, carriers declined on these but improved on cNTB (both compared to placebo); results published: [314] No results—**NCT00325728** No results—**NCT03954899**^a,^^b^
**GABA enhancement **(inc. zolpidem (Ambien), sodium oxybate, allopregnanolone)**—**Increases or modulates GABAergic activity, can promote sleep (see Fig. 4) [315]		
• Optogenetic stimulation of GABAergic interneurons increases autophagy (*i.e.*, Beclin1 and LC3 levels) and reduces Aβ in an AD mouse model [316] • Allopregnanolone enhances autophagy (↑ LC3B-II and ↓ SQSTM1), neuroprotective in a rat glaucoma model [317]; potential synergistic effect of GABAergic modulation on improving sleep and proteostasis in AD • Critically, GABAergic dysfunction is a major part of AD progression; reviewed in [203]	Zolpidem—Insomnia Sodium Oxybate—narcolepsy Allopregnanolone—postpartum depression	**4 (1)** **NCT00814502** – zolpidem CR, AD, or vascular dementia; small sample size limitation (8 zolpidem CR, 9 placebo), no changes or trend in primary outcomes (sleep efficiency and time) **NCT03075241** – Phase 3, zolpidem and zoplicone (separately), insomnia in AD; 81 min increase in nocturnal sleep (zoplicone only), reduced night-time awakenings and length (either zolpidem or zoplicone), compared to placebo; results published: [318] No results—**NCT00706186** No results—**NCT03748303**^b^
**Antihistamines—**Inhibit histaminergic signalling in the tuberomammillary nucleus of the lateral hypothalamus (+ peripheral effects)**;** blocks arousal and promotes SWS, but not REM (see Fig. 4) [18, 319]		
• Could be an effective therapeutic factor in the treatment of AD through the antagonists against H3R and regulation of H2R • Specific mechanism in protein clearance is yet to be investigated [320]	Allergies, insomnia	**3 (0)** **NCT01548287** – Phase 2, AZD5213, sleep in MCI and mild AD; no clear benefit in primary outcome (total sleep time) **NCT01028911** – Phase 1, PF-03654746 and donepezil, mild-to-moderate AD; no serious adverse events, 5/7 participants in treatment group experienced non-serious adverse events No results—**NCT01009255**
**Adrenergic agonist **(dexmedetomidine)**—**Inhibits noradrenergic release (notably in the LC), decreasing arousal [321, 322]		
• Binds pre-synaptic α2 adrenergic receptors—hyperpolarizing LC neurons and ↓ noradrenergic release—↑ EEG slow wave oscillations [321, 322] & ↑ glymphatics [323] • Dexmedetomidine ↑ glymphatic flow—effective adjuvant to improve intrathecal drug delivery in mice [323] • Adrenergic agonism can impact sleep & proteostasis via glymphatics. However, dexmedetomidine is commonly used as an analgesic and sedative, potentially limiting sleep quality	Sedation	No results—**NCT04205539** – no results; withdrawn prior to enrollment - pending COVID-19 pandemic
**mTOR inhibitor **(rapamune)**—**Multiple signalling targets, may modulate sleep in AD through autophagy induction [1]		
• Compared to the other therapies in this table, rapamune induces ubiquitin proteasomal and autophagic-lysosomal systems directly, but not selectively Potential Experimental Use—enhancing proteostasis to reduce proteinopathies that reduce sleep	Immunosuppressant	**1 (1)** No results—**NCT04200911**^b^
**Anti-fatigue **(modafinil)**—**Dopamine reuptake inhibitor: dopamine helps regulate circadian rhythmicity and REM (notably in Parkinson’s disease and REM sleep behavior disorder) [324–326]		
• Modafinil—activates PI3K/Akt/mTOR/P70S6K signalling • Suppresses excessive autophagy and apoptosis of hippocampal neurons as a result of sleep deprivation—rescue of aberrant proteostasis from sleep impairment [327]	Obstructive sleep apnea, narcolepsy	**1 (0)** **NCT00626210** – modafinil; limited results because study was terminated early (low enrollment: 2 participants)
**Cannabis and endocannabinoid system,** i.e. cannabidiol (inc. nabilone)**—**Endocannabinoid system regulates circadian-control of physiological processes (inc. sleep) [328, 329]		
• ↑ Aβ degradation genes ACE1, IDE and ECE1 and heat shock proteins – may ↓ tau and Aβ misfolding • Further research required to understand mechanisms, i modification of phosphorylation pathways, and alterations of glial reactivity or Aβ load [330, 331]	Epilepsy, common usage (*i.e.*, for anxiety, pain)	**4 (3)** **NCT02351882** – Phase 2 and 3, nabilone, moderate-to-severe AD; beneficial effects of nabilone on agitation and neuropsychiatric assessment despite higher sedation; sleep not directly assessed; results published: [332] No results—**NCT04516057**^b^**; NCT04436081**^b^**; NCT05239390**^b^
**Serotoninergic modulators and anti-depressants **(inc. trazodone, nelotanserin, sertraline (Zoloft), escitalopram oxalate)**—**reduces REM sleep and increases wakefulness [171, 333]		
• Serotonergic signaling in conjunction with UPRmt (mitochondrial specific unfolded protein response), facilitates communication of proteotoxic stress from neuronal mitochondria to peripheral tissue and helps establish a mechanistic link between remodeling of mitochondrial function by a biogenic amine and metabolic disturbances seen in neurodegenerative conditions [334] • Potential Experimental Use—role for secretion of serotonin in cell-non-autonomous communication of mitochondrial stress [334] • Trazodone reverses the low protein synthesis rates associated with elevated phosphorylated-eIF2α levels, an effect commonly seen with overactive UPR and in NDDs [275, 276]	Depression, anxiety; Trazodone—off-label for sleep disturbances in AD	**11 (2)** **NCT00895895** – Phase 2, SAM-531, mild-to-moderate AD; no differences in primary outcome (ADAS-Cog), some sporadic significance in secondary outcomes, trend to benefit in NPI (inc. sleep behaviors) at 3 mg dose but not directly tested for sleep **NCT02708186**^a^ – Phase 2, nelotanserin, REM sleep behavioral disorder in DLB/PDD; no differences vs. placebo in REM behavioral disorder-associated movements; results published: [333] **NCT01142258** – Phase 3, trazodone, sleep disorder in AD; Trazodone increased night-time sleep by 42.5 min with no effect on daytime sleepiness or cognition; results published: [335] **NCT00009191** – Phase 4, sertraline, depression in AD; reduced depressive symptoms, sleep not assessed; results published: [336] No results—**NCT01841125; NCT01867775; NCT00519298; NCT02871427; NCT00103649; NCT05282550**^a,b^**; NCT05004987**^b^
**Anti-psychotics **(inc. aripiprazole, risperidone) and** anti-convulsants **(inc. gabapentin enacarbil, levetiracetam)**—**Less sleep related, drowsiness as a side effect; Note: Gabapentin Enarcarbil is a GABA agonist [337]		
• Limited link to proteostasis • Broad neurotransmitter receptor modulation may have non-specific effects as described in more specific rows above	Various uses (*i.e.*, bipolar disorder, schizophrenia, epilepsy, restless legs disorder; anti-convulsant)	**8 (2)** **NCT01438060** – Phase 3, aripiprazole, AD with psychotic symptoms; no obvious changes in NPI sleep score (secondary outcome), no significant change in NPI psychosis (primary outcome) **NCT02002819** – Phase 2, levetiracetam, AD; some cognitive benefits, sleep not a primary or secondary outcome; results published: [338] **NCT00208819**; **NCT02078310; NCT00232570; NCT04341467**^b^ – no results (anti-psychotics) No results—**NCT03790709; NCT03082755**^b^ (anti-convulsants)
**Other dietary supplements** (inc. citicoline, probiotics)—**citicoline –** increased choline levels for acetylcholine synthesis, cholinergic signalling from PPT/LDTT and basal forebrain involved in REM sleep and wakefulness (reviewed in [339]); **probiotics** – likely indirect benefits on sleep from anti-oxidant and anti-inflammatory effects [340]		
• Limited direct relevance to proteostasis	**Citicoline** – potential benefits of supplementation in aging-related cognitive decline [341]; **Probiotics** – various uses	**2 (2)** **NCT05200208**^b^ (citicoline) No results—**NCT05145881**^b^ (probiotics)

Studies indicated as “results available” have results posted on clinicaltrials.gov, or a publication indexed in clinicaltrials.gov by NCT identifier

*AD* Alzheimer’s disease, *ADAS-cog* Alzheimer’s Disease Assessment Scale–Cognitive Subscale, *cNTB* Computerized neuropsychological test battery, *DLB* Dementia with Lewy bodies, *eIF2α* Eukaryotic initiation factor 2α, *GWAS* Genome-wide association study, *IADL* Instrumental activities of daily living, *LC* Locus coeruleus, *PPT/LDT* Pedunculopontine and laterodorsal tegmental nuclei, *MCI* Mild cognitive impairment, *MMSE* Mini-Mental State Examination, *NDD* Neurodegenerative disease, *NPI* Neuropsychiatric Inventory, *PDD* Parkinson’s disease dementia, *PQSI* Pittsburgh Sleep Quality Index, *REM* Rapid eye movement, *SNP* Single-nucleotide polymorphism, *SWS* Slow wave sleep, *UPR* Unfolded protein response

^a^Refers to non-AD studies that were included in the search and considered relevant (MCI, aging, or broad/other dementia)

^b^Refers to ongoing studies with clinicaltrials.gov status “Not yet recruiting”, “Recruiting”, “Enrolling by invitation”, or “Active, not recruiting”

**Table 3:** Non-pharmacological sleep interventions in clinical trials for Alzheimer’s disease

**Intervention**	**Relevance to proteostasis**	**# of AD trials (# ongoing)** **clinicaltrials.gov identifier** – Note if results available
**Auditory and Light Stimulation/Therapy** (inc. GENUS device, Neuro RX Gamma, acoustic stimulation; see Fig. 4)	Entrainment of gamma oscillations (40 Hz visual + auditory stimulation) has been linked to reducing Aβ and tau in AD models [342, 343]; reviewed in [344]. Optogenetic stimulation of parvalbumin inhibitory interneurons leads to a ~ 50% reduction of Aβ in 5xFAD mice, with increased microglial response and uptake [342]. Although a direct linkage to mechanisms of proteostasis is not described, a linkage between neuronal circuitry, in particular GABAergic inhibition, and clearance of Aβ and tau is indicated	**21 (11)** Results available—**NCT00946530; NCT03160027; NCT03357328**^a^ **NCT03484143; NCT04055376**^b^**; NCT00065689; NCT04073628**^b^**; NCT02502045; NCT03328195; NCT03672474; NCT03777722**^b^**; NCT03933696**^b^**; NCT04574921; NCT02686190; NCT04277104**^b^**; NCT05519137**^b^**; NCT05016219**^b^**; NCT05655195**^b^**; NCT05260177**^b^**; NCT05596994**^b^**;** ^b^**NCT05015478**^b^
**Behavioral** (inc. CBT-I, sleep education; see Fig. 4)	Therapy has no direct interaction with proteostasis; however, improvements of proteostasis via behavioral changes would strengthen the sleep-proteostasis linkage. Difficult to model pre-clinically	**21 (13)** Results available—**NCT04533815; NCT00013182; NCT00183378** – No results**—NCT03455569; NCT04100057**^b^**; NCT03954210**^b^**; NCT04779866**^b^**; NCT03840083**^a,b^**; NCT00393627**^a^**;** ^b^**NCT03256539**^b^**; NCT01920672; NCT05309577**^b^**; NCT05452031**^b^**; NCT05565833**^b^**; NCT05102565**^b^**; NCT05138848**^b^**; NCT05555381**^a,b^**; NCT05015803**^b^**; NCT05246332; NCT05050812**^a,b^**; NCT05350410**
**Brain Stimulation** (inc. rTMS, tDCS, DREEM headband)	Broadly, neuronal stimulation can mitigate electrophysiological dysfunction and excitatory-inhibitory imbalance that occurs in AD [345]. On top of promoting cognition and sleep, depending on the targeted brain regions/networks, balancing circuits in AD may reduce neuronal activity-mediated Aβ and tau spread	**10 (5)** Results available—**NCT01894620** No results**—NCT04122001**^b^**; NCT04570761; NCT03270137; NCT04855630**^b^**; NCT03670615; NCT05544201**^b^**; NCT05102045; NCT05200897**^b^**; NCT05715866**
**CPAP** (and related devices; 6); **upper airway sleep surgery** (1); **HBOT** (1)	Therapy has no direct interaction with proteostasis; improved sleep quality theoretically would improve proteostasis mechanisms in AD patients	**8 (4)** Results available—^a^**NCT01482351; NCT01962779** No results**—NCT04905238**^b^**; NCT03929302; NCT02474251; NCT05094271**^b^**;** ^a,b^**NCT05433883** (surgery)**; NCT05349318**^b^ (HBOT)
**Music Therapy** (see Fig. 4)	Therapy has no direct interaction with proteostasis; improved sleep quality theoretically would improve proteostasis mechanisms in AD patients	**4 (1)** Results available—**NCT04157244** No results**—NCT04809545**^a^**; NCT04327778; NCT05309369**^b^

Studies indicated as “Results available” have results posted on clinicaltrials.gov, or a publication indexed in clinicaltrials.gov by NCT identifier

See clinicaltrials.gov pages for detailed study details and results

*AD* Alzheimer’s disease, *CBT-I* Cognitive Behavioral Therapy for Insomnia, *CPAP* Continuous positive airway pressure, *HBOT* Hyperbaric oxygen therapy, *rTMS* Repetitive transcranial magnetic stimulation, *tDCS* Transcranial direct current stimulation

^a^Refers to non-AD studies that were included in the search and considered relevant (MCI, aging, or broad/other dementia)

^b^Refers to ongoing studies with clinicaltrials.gov status “Not yet recruiting”, “Recruiting”, “Enrolling by invitation”, or “Active, not recruiting”

#### Trazodone

Trazodone hydrochloride is a unique therapy on this list due to hitting multiple targets on the sleep-proteostasis axis. Trazodone is an antidepressant therapy approved for use in major depressive disorder, and is commonly used for off-label treatment of patients with sleep disorders, and in AD. Multiple neurotransmitter systems are affected by trazodone treatment, including selective agonism and antagonism of serotonin receptors, serotonin transporter inhibition and reuptake inhibition, antagonism of histaminergic and α1 and α2 adrenergic receptors, and, to a lesser degree, trazodone exhibits anticholinergic activity (reviewed in [346]). In sum, the neuromodulatory effects of trazodone can shift neuronal control of the sleep–wake balance towards sleep (Figs. 2 and 4).

In a phase 3 clinical trial, AD patients with sleep disturbances were treated with 50 mg of trazodone once per day before bed over a 2-week period. Treatment significantly increased sleep time and trended to reduced arousals and wake periods throughout the night, without exacerbating daytime napping or EDS. With this short-term treatment, cognition was unaffected ([335]; Table 2). However, evidence suggests long-term recorded trazodone use (median dose: 50 mg/day) in AD patients with sleep disturbances slows cognitive decline on mini-mental state examination, over a 4-year follow-up [347]. At a higher dose of 200 mg, trazodone is safe and feasible in AD patients, and is within the predicted range for regulating UPR (reviewed in [276, 348]). Although the mechanism of the trazodone-UPR interaction is through normalizing protein synthesis rates [276], Aβ and tau degradation and clearance may be indirectly improved through mitigating neurodegenerative burden, and reducing UPR overactivation which will improve cellular homeostasis (and proteostasis).

Further work is necessary to identify how trazodone can impact AD progression, and at what dose, including potential benefits on protein clearance and neuronal function. Currently, a phase 2 clinical trial of trazodone in MCI is recruiting (as of February 2023) to test the effect of a 4-week, 50 mg treatment on SWS quantity and quality, sleep onset and fragmentation, cognition, as well as hippocampal neuronal activity by functional magnetic resonance imaging (ClinicalTrials.gov Identifier: NCT05282550; Table 2).

#### Adrenergic alpha receptor agonism

Agonists of adrenergic α receptors impact the sleep–wake cycle via a reduction in locus coeruleus noradrenergic activity, decreasing arousal and promoting sleep [349]. Dexmedetomidine is an α2 adrenergic receptor agonist that has demonstrated benefit as a therapeutic in preclinical in vitro and in vivo models including anti-inflammatory, pro-cognitive and pro-neurotrophic effects [350–354], despite association with increased tau phosphorylation (*i.e.*, at pS202, pT205, pT231, pS396) in cells and rodents lasting up to 6-h post treatment [355, 356]. Dexmedetomidine is used clinically as an analgesic and sedative, due to an induction of NREM stage 2-to-3 sleep, including increased slow wave delta oscillations and spindles in EEG [321–323]. One potential benefit to the sleep-proteostasis interaction is that dexmedetomidine acutely enhances glymphatics [323] (see Table 2), but its usage as a primary therapeutic for AD remains limited and the potential for exacerbating tau pathology is of concern.

#### Orexin antagonism – Suvorexant

Currently, the most promising sleep modulating therapy in AD is the orexin antagonist suvorexant, which is used in the treatment of insomnia [304] and was approved in February 2020 for treatment of sleep disorder symptoms in AD patients. Orexin signaling is also reported to regulate cognition, and in AD there is orexinergic dysfunction that occurs with elevated CSF orexin [184, 187]. Suvorexant competes with orexin for binding to receptors OXR1 and OXR2, promoting sleep [304, 357, 358]. In a phase 3 trial to treat insomnia with mild-to-moderate AD, 4 weeks of daily oral treatment with suvorexant significantly improved total sleep time by 73 min more than baseline, (45 min in the placebo group); and treatment did not worsen cognition and was well-tolerated [308]. Recently, Lucey and colleagues demonstrated that acute suvorexant treatment in healthy middle-aged adults decreased CSF levels of Aβ by 10–20% and p-tau (T181) by 10–15%, but not at other p-tau isoforms – S202 or T217 [359].

Suvorexant may prove beneficial in combination therapies, potentially with the recently approved aducanumab or lecanemab, and to probe the sleep-proteostasis axis. Promoting sleep with suvorexant in preclinical AD models can be utilized to assess alterations in specific neuronal vulnerability to tau and Aβ, and potential improvements in glymphatic clearance and autophagic flux. Modulation of orexinergic function, although limited to a small cellular population, may exert beneficial network effects throughout the brain by promoting sleep, helping to balance circadian arrhythmicity, and therefore mitigating deleterious changes in proteostasis. Rebalancing specific neuronal population function (*i.e.,* orexinergic) can potentially correct a network-wide dysfunction seen in AD; for example, suvorexant treatment enhances hippocampal long term potentiation in AD model mice [360].

Characterizing proteostasis improvement from suvorexant treatment in sleep- and AD-associated neurons and regions, in hypothalamic orexin neurons, as well as EC-hippocampal cognitive circuits (Fig. 4), will help define the sleep and proteostasis interaction across disease progression, and inform efficacy of treatment based off disease staging.

## Conclusions

As supported by evidence for early disruptions in both mechanisms, we postulate that experiments probing the sleep-proteostasis axis will be critical in understanding AD progression and associations with age. Future work will elucidate novel markers of prodromal AD, including PET and plasma biomarkers for neuronal vulnerability to proteostasis alterations, and EEG disruptions indicative of sleep disruption. Furthermore, single, and combinatorial therapies can be tested against sleep and proteostasis, including how their efficacy varies across AD progression. It is important to note that sleep impairments often coincide with hypothalamic–pituitary–adrenal axis activity and behavioral stress, which also exert effects on cellular proteostasis [361, 362]; reducing stress should be considered in the design and interpretation of preclinical sleep deprivation paradigms [363], to mitigate additional stress-related alterations in proteostasis.

There are major challenges facing this research field; notably, the technical challenge of collecting and analyzing molecular-, cellular-, circuit- and behavioral-level data, for determining neuronal-, regional- and temporal-vulnerabilities to sleep and proteostasis deficits. Bioinformatic approaches will be necessary to pinpoint critical interactions of these pathologies, and early predictors of AD progression and cognitive decline. Once we understand the sleep and proteostasis interaction, the next challenge is translatability of novel or repurposed sleep- and proteostasis-targeted therapies. However, with the recent successes of suvorexant (ClinicalTrials.gov Identifier: NCT02750306; [308]), aducanumab (ClinicalTrials.gov Identifier: NCT02477800 and NCT02484547) and lecanemab (ClinicalTrials.gov Identifier: NCT01767311 and NCT03887455), there is high potential for successful targeted and personalized therapies promoting sleep and protein clearance in AD.

Future experiments will facilitate creation of a framework for disease-modifying treatments in AD to slow or halt this proteinopathy and reverse behavioral deficits, including understanding potential therapeutic efficacy at different stages of disease progression. Probing neuronal electrophysiology and sleep patterns will allow identification and repurposing of therapies targeting these mechanisms and their interaction with proteostasis, and thereby facilitate novel drug discovery. Sleep impairments, proteinopathy and proteostasis failure are common occurrences with aging and in NDD, and therefore the conclusions raised herein pertain to a broad array of brain disorders, including cases of mixed pathologies.

## Data Availability

Not applicable.

## Abbreviations

ATF6: Activating transcription factor-6
AD: Alzheimer’s disease
APP: Amyloid precursor protein
ApoE4: Apolipoprotein E4
AQP4: Aquaporin-4
ALP: Autophagic-lysosomal pathway
Aβ: β-Amyloid
BiP: Binding immunoglobulin protein
BF: Basal forebrain
BBB: Blood-brain barrier
C9orf72: Chromosome 9 open reading frame 72
CHOP: CCAAT/enhancer binding protein homologous protein
CSF: Cerebrospinal fluid
DLB: Dementia with Lewy bodies
DRN: Dorsal raphe nucleus
EEG: Electroencephalogram
ER: Endoplasmic reticulum
EAL: Endosome-autophagosome-lysosome
EC: Entorhinal cortex
eIF2α: Eukaryotic initiation factor 2α
EDS: Excessive daytime sleepiness
FTD: Frontotemporal dementia
FUS: Fused in sarcoma
Htt: Huntingtin
HD: Huntington’s disease
IRE1: Inositol-requiring enzyme 1α
ISF: Interstitial fluid
LATE: Limbic-predominant age-related TDP-43 encephalopathy
LC: Locus coeruleus
MCH: Melanin-concentrating hormone
MCI: Mild cognitive impairment
NDD: Neurodegenerative disease
NREM: Non-rapid eye movement
PD: Parkinson’s disease
PPT/LDT: Pedunculopontine and laterodorsal tegmental nuclei
POA: Preoptic area
PSQI: Pittsburgh sleep quality index
PERK: Protein kinase RNA-like ER kinase
PET: Positron emission tomography
REM: Rapid eye movement
RBD: REM sleep behavior disorder
SST: Somatostatin
SWS: Slow wave sleep
SCN: Suprachiasmatic nucleus
TDP-43: TAR DNA-binding protein 43
TRN: Thalamic reticular nucleus
UPS: Ubiquitin proteasome system
UPR: Unfolded protein response
VIP: Vasoactive intestinal polypeptide
VLPO: Ventrolateral preoptic area
